# FIP200 controls the TBK1 activation threshold at SQSTM1/p62-positive condensates

**DOI:** 10.1038/s41598-021-92408-4

**Published:** 2021-07-05

**Authors:** David Schlütermann, Niklas Berleth, Jana Deitersen, Nora Wallot-Hieke, Olena Friesen, Wenxian Wu, Fabian Stuhldreier, Yadong Sun, Lena Berning, Annabelle Friedrich, María José Mendiburo, Christoph Peter, Constanze Wiek, Helmut Hanenberg, Anja Stefanski, Kai Stühler, Björn Stork

**Affiliations:** 1 Institute of Molecular Medicine I, Medical Faculty, University Hospital Düsseldorf, Heinrich Heine University Düsseldorf, Universitätsstr. 1, Building 22.03, 40225 Düsseldorf, Germany; 2 Department of Otorhinolaryngology and Head/Neck Surgery, Medical Faculty, University Hospital Düsseldorf, Heinrich Heine University Düsseldorf, 40225 Düsseldorf, Germany; 3 Department of Pediatrics III, University Hospital Essen, University of Duisburg-Essen, 45122 Essen, Germany; 4 Molecular Proteomics Laboratory, Biologisch-Medizinisches Forschungszentrum (BMFZ), Heinrich Heine University Düsseldorf, 40225 Düsseldorf, Germany

**Keywords:** Cell biology, Autophagy, Cell signalling

## Abstract

The protein kinase TBK1 is a central regulator of innate immune responses and autophagy, and ablation of either function has been linked to neuroinflammatory or degenerative diseases. Autophagy is an intracellular process that recycles old or damaged proteins and organelles. In recent years, the TBK1-dependent regulation of autophagy pathways has been characterized. However, the autophagy-dependent regulation of TBK1 activity awaits further clarification. Here, we observed that TBK1 is recruited to SQSTM1/p62-containing aggregates via the selective autophagy receptor TAX1BP1. In these aggregates, TBK1 phosphorylates SQSTM1/p62 at serine 403 and thus presumably regulates the efficient engulfment and clearance of these structures. We found that TBK1 activation is strongly increased if FIP200, a component of the autophagy-inducing ULK1 complex, is not present or cannot bind to TAX1BP1. Given our collective findings, we hypothesize that FIP200 ensures the inducible activation of TBK1 at SQSTM1/p62 condensates.

## Introduction

(Macro-)autophagy is an intracellular recycling process that maintains cellular homeostasis by degrading old or damaged proteins and organelles. Autophagy can be either nonselective or selective with regard to its cargo. Nonselective autophagy occurs at basal levels in most cell types but is also inducible under conditions of nutrient depletion. The process is initiated with the nucleation of a phagophore, which engulfs bulk cargo and expands into double-membraned vesicles called autophagosomes. Autophagosomes transport the cargo to lysosomes, in which the cargo is ultimately degraded. The initiation of autophagic processes is centrally regulated by two kinase complexes: (1) the UNC-51-like autophagy activating kinase 1 (ULK1) protein kinase complex containing the Ser/Thr kinase ULK1 and the adapter proteins autophagy-related (ATG) 13, ATG101 and focal adhesion kinase (FAK)-interacting protein of 200 kDa (FIP200); and (2) the class III phosphatidylinositol 3-kinase (PtdIns3K) lipid kinase complex containing the phosphatidylinositol 3-kinase catalytic subunit type 3 (PIK3C3/VPS34) and the associated proteins phosphoinositide 3 kinase regulatory subunit 4 (PIK3R4/VPS15), Beclin 1, ATG14, and nuclear receptor-binding factor 2 (NRBF2)^1,2^. Both kinase complexes are also involved in the regulation of selective autophagy, during which the cargo is specifically targeted by selective autophagy receptors (SARs)^3,4^. Selective autophagic processes are classified based on the type of degraded cargo, such as damaged mitochondria (mitophagy), protein aggregates (aggrephagy), or intracellular pathogens (xenophagy)^3^. The SARs sequestosome 1 (SQSTM1/p62), neighbor of *BRCA1* gene 1 (NBR1), nuclear dot protein 52 kDa (NDP52), Tax1 binding protein 1 (TAX1BP1), and optineurin (OPTN) all belong to the SQSTM1/p62-like receptor (SLR) family and represent the best-studied family of SARs^5^. They simultaneously bind ubiquitin moieties on the cargo and phosphatidylethanolamine (PE)-conjugated ATG8 family proteins^5^, which are attached to the membrane of forming autophagosomes^6^. Recently, NDP52 was shown to attract the ULK1 complex to damaged mitochondria^7^ or cytosolic pathogens^8^ by binding to a C-terminal region of FIP200. Once recruited, ULK1 initiates the formation of the phagophore directly at the cargo^9^. The recruitment of the ULK1 complex is facilitated by TANK-binding kinase 1 (TBK1)^7,8^. The Ser/Thr kinase TBK1 is a central regulator of innate immune responses, but in recent years, its involvement in autophagy signaling pathways has been discovered. In addition to facilitating recruitment of the ULK1 complex to mitochondria or intracellular pathogens, TBK1 directly regulates other components of the autophagy signaling cascade, including AMP-activated protein kinase (AMPK)^10^, syntaxin 17^11^, and various SARs^7,8,12–17^. TBK1-dependent phosphorylation frequently modulates the binding affinities of SARs; for example, it has been reported that TBK1-catalyzed phosphorylation of SQSTM1/p62 at Ser403 enhances ubiquitin binding^13,14,18^.

The innate immune response is the first line of defense during viral infections. Pattern recognition receptors (PRRs) detect specific bacterial or viral components, ultimately inducing type I interferon (IFN) and proinflammatory cytokine production to prevent viral invasion and replication^19^. PRRs include Toll-like receptors (TLRs), retinoic acid-inducible gene I (RIG-I)-like receptors (RLRs), and cytosolic DNA receptors such as cyclic GMP-AMP synthase (cGAS). Upon ligand binding, TLR3/4, RIG-I, and cGAS mediate the recruitment of TBK1 to the adaptor proteins TIR domain-containing adapter-inducing interferon-β (TRIF), mitochondrial antiviral signaling protein (MAVS), and stimulator of interferon genes (STING), respectively^20^. Local clustering leads to trans-autophosphorylation at Ser172 and thus activation of TBK1^21,22^. Once activated, TBK1 phosphorylates interferon regulatory factor (IRF) 3 and IRF7, thereby inducing their dimerization and nuclear translocation. In the nucleus, IRF3 and IRF7 activate type I IFN gene expression^23^. TLR signaling, e.g., binding of the adaptor proteins TRIF and myeloid differentiation primary response 88 (MyD88) to Beclin 1, also induces autophagy^24,25^. On the other hand, autophagy can exert anti-inflammatory effects^26^, such as by targeting the RIG-I-MAVS axis^27^ or via TAX1BP1-mediated selective degradation of TRIF^28^. Furthermore, TAX1BP1 and the ubiquitin-editing enzyme A20 target TBK1 to inhibit the immune response by disrupting the TRAF3-TBK1 signaling complex^29^.

Mutations of the *TBK1* gene are connected to several diseases, including childhood herpes simplex encephalitis (HSE), amyotrophic lateral sclerosis (ALS), frontotemporal dementia (FTD), and normal-tension glaucoma (NTG) (reviewed in^30^). These diseases are caused either by dysregulated autophagy or by impaired IFN production^30^. Loss-of-function mutations in the *TBK1* gene have been linked to ALS and FTD^31,32^. In turn, gain-of-function *TBK1* mutations (i.e., gene duplication) have been reported in patients with NTG. Therefore, TBK1 must be tightly regulated to maintain cellular homeostasis. Although the involvement of TBK1 in the regulation of autophagy is known, the regulation of TBK1 itself during autophagic processes awaits further clarification.

In this study, we found that TBK1-TAX1BP1-SQSTM1/p62 aggregates develop in cells with autophagy impairment in general and FIP200 deficiency in particular. TBK1 is activated in these aggregates and phosphorylates SQSTM1/p62 at Ser403. These aggregates are likely caused by proteotoxic stress induced by inhibition of autophagy. The recruitment of TBK1 to these aggregates is mediated by TAX1BP1. The activation of TBK1 is clearly increased in cells if FIP200 is not present or cannot bind to TAX1BP1, indicating that FIP200—in addition to recruiting the downstream autophagy machinery—inhibits aberrant activation of TBK1. We propose that FIP200 controls the TBK1 activation threshold and ensures the inducibility of TBK1 activity.

## Results

### Loss of autophagy in general and loss of components of the ULK1 complex in particular lead to increased focal accumulation and activation of TBK1

**Figure 1:**
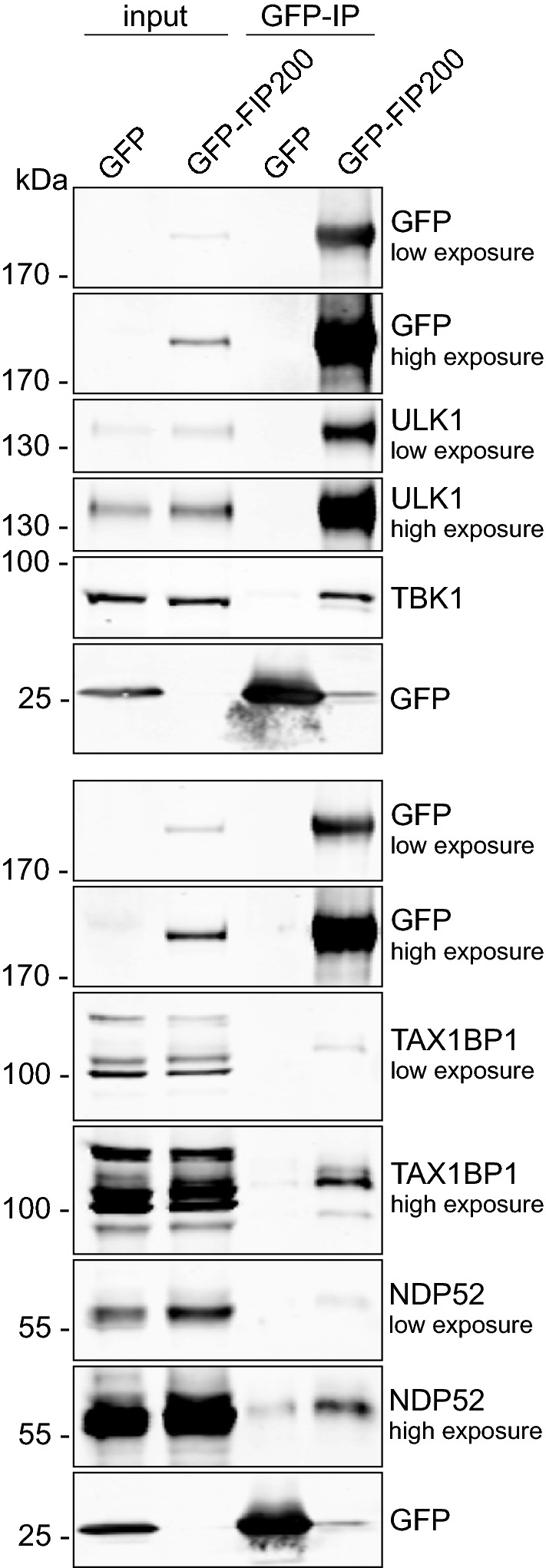
FIP200 interacts with TBK1. Flp-In T-REx 293 cells inducibly expressing GFP or GFP-FIP200 were treated with 0.1 µg/ml doxycycline for 16 h to induce expression. Afterwards, the GFP proteins were purified overnight using GFP-trap beads. Cleared cellular lysates (input) and purified proteins were immunoblotted for the indicated proteins. The full-length blots are presented in Figure S5.

To search for novel FIP200-interacting proteins, we performed mass spectrometry (MS) using GFP-FIP200 as bait. Among the identified binding partners were TBK1, TBKBP1/SINTBAD, and several autophagy receptors (TAX1BP1, SQSTM1/p62, and NBR1) (Table S1). The interaction between GFP-FIP200 and TBK1 was confirmed by immunopurification (Fig. 1). Recent reports have shown that TBK1 binds to the autophagy receptor NDP52 and facilitates ULK1 complex recruitment via FIP200^7,8^. In our immunopurification experiments, we observed that GFP-FIP200 bound to the autophagy receptors TAX1BP1 and NDP52 (Fig. 1). Accordingly, we assumed that FIP200 interacts with TBK1 via TAX1BP1 and/or NDP52. Since the cells used for MS and immunopurification were cultured in complete medium, it is likely that these interactions are constitutive.

**Figure 2:**
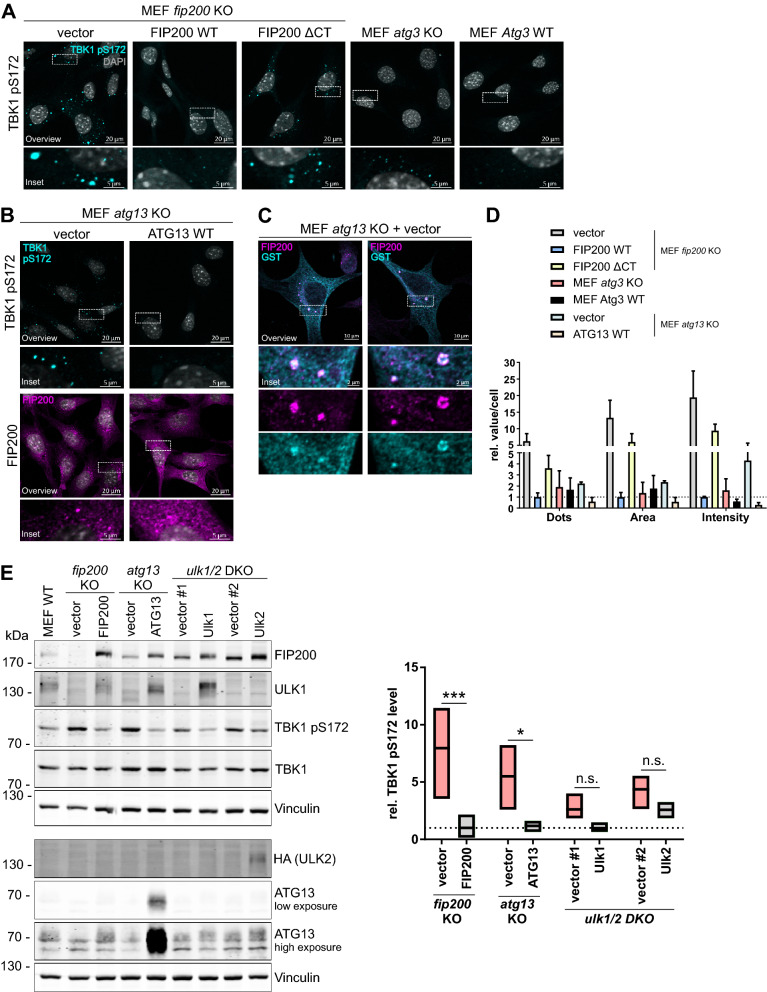
Loss of components of the ULK1 complex leads to aberrant accumulation and increased activation of TBK1. (**A**) *fip200* KO MEFs were transfected with an empty vector or cDNA encoding full-length FIP200 (FIP200 WT) or C-terminally truncated FIP200 (amino acids 1369–1594, FIP200 ΔCT). These transfectants, *atg3* KO and *Atg3* WT MEFs were fixed in 4% PFA and immunostained for TBK1 pS172. Representative sections are depicted. (**B**) *atg13* KO MEFs transfected with an empty vector or cDNA encoding WT ATG13 were fixed in 4% PFA and immunostained for TBK1 pS172 and FIP200. Representative sections are depicted. (**C**) For costaining of TBK1 and FIP200, *atg13* KO MEFs were transiently transfected with cDNA encoding GST-TBK1, fixed in 4% PFA, and immunostained for FIP200 and GST. Representative sections are depicted. For IF imaging, DAPI was used to stain nuclei. (**D**) The number, area, and intensity of TBK1 pS172-positive structures in at least 198 cells per cell line were quantified using ImageJ 1.53c and normalized to the values in FIP200 WT cells. The means + SDs of three independent experiments are shown. (**E**) Cleared cellular lysates of MEF *ulk1/2* DKO, *fip200* KO and *atg13* KO MEFs transfected with an empty vector or the respective WT cDNA (encoding FIP200, ATG13, ULK1, or ULK2) were immunoblotted for the indicated proteins. The full-length blots are presented in Figure S6. The densities of the bands on immunoblots from three independent experiments were quantified and normalized to those of Vinculin. The normalized densities of all samples of each cell line were then normalized to that of the respective reconstituted cell line. Only the relative levels of TBK1 pS172 are shown. The boxes represent the highest and the lowest value, while the centerline shows the mean. Statistical analysis was performed using two-way ANOVA with uncorrected Fisher's LSD test. n.s. = not significant; *p < 0.05; ***p < 0.001.

It has been previously reported that the C terminus of FIP200 mediates binding to the autophagy receptor(s) NDP52 and/or SQSTM1/p62^7,8,33^. The interaction between the C terminus of FIP200 (amino acids 1352–1441) and NDP52 has been shown to be mandatory for the recruitment of the ULK1 complex to damaged mitochondria or intracellular bacteria, respectively^7,8^, whereas the binding of the C-terminal FIP200 claw domain (amino acids 1494–1594) to SQSTM1/p62 promotes autophagosome formation at SQSTM1/p62-ubiquitin condensates^33^. To analyze the potential role of FIP200 and, in particular, its C terminus in TBK1 activation, we made use of *fip200* knockout (KO) mouse embryonic fibroblasts (MEFs) that were transfected with an empty vector or cDNA encoding wild-type FIP200 or a variant lacking the C-terminal part of FIP200 (amino acids 1369–1594, ΔCT). Notably, we observed that loss of FIP200 led to aberrant accumulation of activated TBK1, as determined by immunofluorescence (IF) using antibodies specific for TBK1 phosphorylated at Ser172 (pS172) (Fig. 2A). This finding is in accordance with those of a study by Goodwin et al. investigating ferritinophagy^34^. Expression of wild-type FIP200 in *fip200* KO MEFs blocked the accumulation of phospho-TBK1. In contrast, the C-terminally truncated version of FIP200 reduced the focal localization of phospho-TBK1 but was not able to completely prevent it (Fig. 2A). Since autophagy is blocked in FIP200-deficient MEFs^35^, we also investigated the influence of generally defective autophagy on TBK1 regulation. For this, we examined the phosphorylation status of TBK1 in MEFs deficient in ATG3 via immunoblotting and IF (Fig. 2A, Figure S1A). ATG3 mediates the E2-like conjugation of LC3 to PE, which is an essential step during autophagy^6^. *Atg3* KO cells showed greater TBK1 activation than wild-type MEFs (Fig. 2A, Figure S1A). We observed that blockade of autophagy with the lysosomal V-ATPase inhibitor bafilomycin A_1_ (BafA_1_) increased TBK1 activation in wild-type cells but not in *atg3* KO cells (Figure S1A). Next, we investigated whether depletion of ATG13, another component of the ULK1 complex, affects TBK1 activation. IF analysis of *atg13* KO cells revealed that both TBK1 and FIP200 formed puncta (Fig. 2B). Transient expression of GST-TBK1 showed that the observed TBK1 and FIP200 puncta colocalized, and partially circular structures were detectable (Fig. 2C). To enable better comparison, the phospho-TBK1-positive puncta per cell were counted in all mentioned cell lines, and the size and intensity of each punctum were determined (Fig. 2D). Loss of FIP200 led to an increase in the number of puncta per cell. Moreover, the sizes and intensities of these phospho-TBK1-positive structures were increased. Removal of the FIP200 C-terminal domain increased the number of TBK1 aggregates by a factor of 3. The sizes and intensities of the resulting structures were also increased (by ~ 5 × per cell and ~ 9 × per cell, respectively) (Fig. 2D). In *atg13* KO cells, the intensity of the phospho-TBK1 puncta was slightly increased, but the number and area were not as high as those in *fip200* KO MEFs. Similarly, there were fewer phospho-TBK1 aggregates in *atg3* KO cells than in *fip200* KO MEFs, and the phospho-TBK1 aggregates in *atg3* KO cells were smaller and less intense than those observed in *fip200* KO MEFs (Fig. 2D).

Next, we investigated TBK1 activation in cells deficient for single components of the ULK1 complex by immunoblotting. Briefly, MEFs deficient in either ATG13 or ULK1/ULK2 were transfected with cDNA encoding wild-type ATG13, ULK1, or ULK2. As determined by immunoblotting, loss of ATG13 increased TBK1 activation, while loss of ULK1/2 led to only a slight increase in TBK1 activation. The absence of FIP200 had the strongest effect on TBK1 activation (Fig. 2E). Next, we analyzed whether FIP200 also influences TBK1 activation in human cell lines by knocking down FIP200 levels in HeLa cells or differentiated THP-1 cells with siRNA. Knockdown of FIP200 with three different siRNAs increased TBK1 activation in both cell lines, indicating the general validity of our observations (Figure S1B). The immunoblots for Flp-In T-REx 293 cells and MEFs (Figs. 1 and 2E) suggested that overexpression of FIP200 does not affect TBK1 protein levels, and this was confirmed by quantification (Figure S1C). Collectively, these data suggest that autophagy in general and the ULK1 complex in particular are important for the regulation of TBK1 activation. Within the ULK1 complex, FIP200 seems to be the main effector controlling TBK1 activation.

### Phospho-TBK1 aggregates are positive for the autophagy receptors TAX1BP1 and SQSTM1/p62

**Figure 3:**
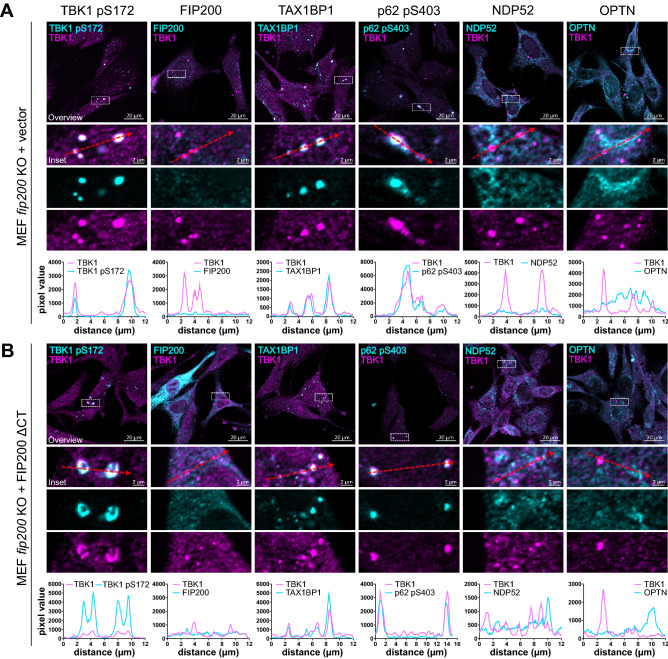
TBK1 aggregates are positive for the autophagy receptors TAX1BP1 and SQSTM1/p62 pS403. (**A** + **B**) *fip200* KO MEFs transfected with an empty vector (**A**) or cDNA encoding FIP200 ΔCT (**B**) were fixed in 100% MeOH and immunostained for TBK1 in combination with TBK1 pS172, FIP200, SQSTM1/p62 pS403, NDP52, or OPTN. Representative sections of three independent experiments are depicted. The bar graphs represent the pixel intensities of the areas indicated by the respective dashed red arrows shown in the insets.

We observed that loss of FIP200 or deletion of its C terminus resulted in aberrant TBK1 accumulation. Normally, protein aggregates are cleared by aggrephagy. Since autophagy receptors recognize the cargoes during selective autophagy and connect them to the autophagy machinery, we analyzed whether the receptors TAX1BP1, NDP52, OPTN and SQSTM1/p62 localize to TBK1 aggregates. As determined by IF, TAX1BP1 and SQSTM1/p62 colocalized with TBK1 aggregates caused by loss of FIP200, while NDP52 and OPTN did not (Fig. 3A). Similar observations were made for cells expressing the C-terminally truncated version of FIP200 (Fig. 3B). It has previously been reported that TBK1 phosphorylates SQSTM1/p62 at Ser403 and that this phosphorylation ensures efficient autophagosomal engulfment of ubiquitinated mitochondria^13^. Phospho-SQSTM1/p62 was highly abundant within the TBK1-TAX1BP1-SQSTM1/p62 aggregates (Fig. 3A,B). We also analyzed whether the C-terminally truncated variant of FIP200 was recruited to TBK1 aggregates. The expressed FIP200 ΔCT showed a cytosolic distribution and no colocalization with TBK1 aggregates (Fig. 3B). Additionally, some of the TBK1-positive aggregates that formed because of FIP200 loss were positive for the autophagy marker LC3, while others were not. Similar observations were made for cells expressing FIP200 ΔCT (Figure S2A). It has been previously reported that the Golgi apparatus acts as a platform for TBK1 activation^36^. Thus, we investigated whether TBK1 aggregates occur in specific organelles. Antibodies against ERGIC-53/p58, ERp72 and Golgin97 were used to stain the endoplasmic reticulum (ER)-Golgi intermediate compartment (ERGIC), the ER, and the Golgi complex, respectively. As determined by IF, TBK1 aggregates did not colocalize with any of these organelles (Figure S2B). Taken together, these data suggest that TBK1 accumulates together with TAX1BP1 and SQSTM1/p62 in large aggregates/condensates when binding to FIP200 is abolished.

### TBK1 aggregation depends on TAX1BP1

**Figure 4:**
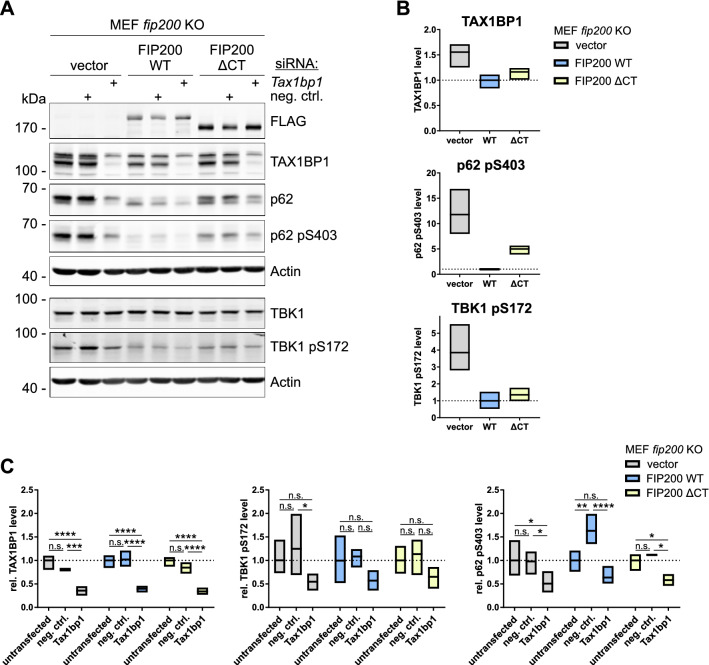
Aberrant TBK1 accumulation depends on TAX1BP1. (**A**) *fip200* KO, FIP200 WT, and FIP200 ΔCT MEFs were transfected with either 20 nM negative control siRNA or 20 nM *Tax1bp1* siRNA for 72 h, and cleared cellular lysates were immunoblotted for the indicated proteins. The FIP200 cDNAs used for reconstitution of *fip200* KO MEFs encoded an N-terminal FLAG peptide. The densities of the bands on immunoblots from three independent experiments were quantified and normalized to those of Actin. The full-length blots are presented in Figure S7. (**B**) To compare basal expression levels between the cell lines used, the normalized densities of the untransfected samples were normalized to the signal of FIP200 WT MEFs. (**C**) To analyze the influence of siRNA transfection, the normalized densities of all samples of each cell line were normalized to that of the corresponding untransfected sample. Only the relative levels of TAX1BP1, TBK1 pS172, and SQSTM1/p62 pS403 are shown. The boxes represent the highest and the lowest value, while the centerline shows the mean. Two-way ANOVA with uncorrected Fisher's LSD test was used to determine differences between treatments. n.s. = not significant; *p < 0.05; **p < 0.01; ***p < 0.001; ****p < 0.0001.

Since we observed that TAX1BP1 colocalizes with TBK1 aggregates, we next investigated whether TBK1-SQSTM1/p62 aggregate formation itself depends on TAX1BP1. Specifically, we decreased TAX1BP1 expression via RNA interference (RNAi) in *fip200* KO, FIP200 wild-type (WT) and FIP200 ΔCT cells and analyzed TBK1 activation and SQSTM1/p62 phosphorylation at Ser403 by immunoblotting. Although we clearly detected focal localization of TBK1 in FIP200 ΔCT cells (Fig. 2A,D), the pS172 signal detected by immunoblotting resembled that of FIP200 WT cells. Knockdown of TAX1BP1 decreased TBK1 activation and SQSTM1/p62 phosphorylation in all cell lines (Fig. 4A–C). Interestingly, we observed that SQSTM1/p62 migrated at a higher molecular weight in *fip200* KO MEFs and in cells expressing the C-terminally truncated FIP200 variant than in WT cells, presumably reflecting increased phosphorylation of SQSTM1/p62, albeit not necessarily only at Ser403 (Fig. 4A). These results indicate that TBK1-SQSTM1/p62 aggregate formation and the phosphorylation of SQSTM1/p62 at Ser403 in the aggregates depend on the autophagy receptor TAX1BP1.

### Impaired autophagy in fip200 KO MEFs contributes to aberrant TBK1 aggregation and activation

**Figure 5:**
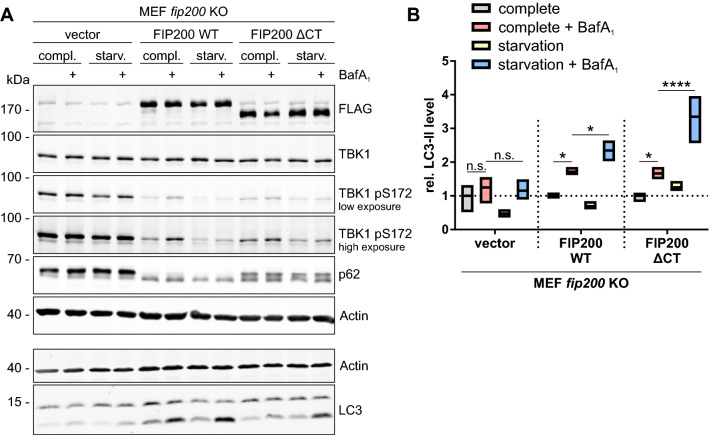
The C terminus of FIP200 is dispensable for starvation-induced autophagy. (**A**) *fip200* KO, FIP200 WT, and FIP200 ΔCT MEFs were cultured in complete or starvation (EBSS) medium in either the absence or presence of 10 nM BafA_1_ for 2 h. Cleared cellular lysates were immunoblotted for the indicated proteins. The FIP200 cDNAs used for reconstitution of *fip200* KO MEFs encoded an N-terminal FLAG peptide. The full-length blots are presented in Figure S8. (**B**) The densities of the bands on immunoblots from three independent experiments were quantified and normalized to those of Actin. The normalized densities of all samples of each cell line were then normalized to that of the respective untreated control sample. Only the relative levels of LC3-II are shown. The boxes represent the highest and the lowest value, while the centerline shows the mean. Two-way ANOVA with uncorrected Fisher's LSD test was used to determine differences between treatments. n.s. = not significant; *p < 0.05; ****p < 0.0001.

Since defective autophagy increased TBK1 activation in *atg3* KO MEFs (Figure S1A), we next investigated whether nonfunctional autophagy was the only mechanism leading to increased TBK1 activation in *fip200* KO MEFs by analyzing starvation-induced bulk autophagy. We performed an LC3 turnover assay in *fip200* KO, FIP200 WT and FIP200 ΔCT-expressing cells. Upon autophagy induction, lipidated LC3-II is increasingly generated and then degraded in autolysosomes^37^. The V-ATPase inhibitor BafA_1_ blocks lysosomal degradation, leading to accumulation of LC3-II^37^. In *fip200* KO MEFs, we did not observe LC3-II accumulation under either normal or starvation conditions, confirming that autophagy signaling was defective. However, expression of FIP200 WT and FIP200 ΔCT restored basal and starvation-induced autophagy (Fig. 5A,B). Similar to the case in *Atg3* WT cells (Figure S1A), TBK1 activation was increased in FIP200 WT and FIP200 ΔCT MEFs after inhibition of autophagy with BafA_1_. Additionally, starvation led to reduced SQSTM1/p62 levels and decreased TBK1 phosphorylation. However, TBK1 and SQSTM1/p62 phosphorylation was increased in *fip200* KO cells and was unaffected by all treatment conditions (Fig. 5A). FIP200 ΔCT cells also resembled FIP200 WT cells during bulk autophagy with regard to TBK1 phosphorylation (see also Fig. 4A), whereas FIP200 ΔCT cells represented an intermediate status between *fip200* KO and FIP200 WT cells with regard to SQSTM1/p62 phosphorylation. Collectively, these findings indicate that the TBK1 accumulation in FIP200 ΔCT cells detected by IF (Fig. 2A) was not solely attributable to impaired autophagy signaling, since bulk autophagic flux appeared rather normal in these cells.

### Loss of FIP200 further enhances TBK1 accumulation and activation caused by defective autophagy

**Figure 6:**
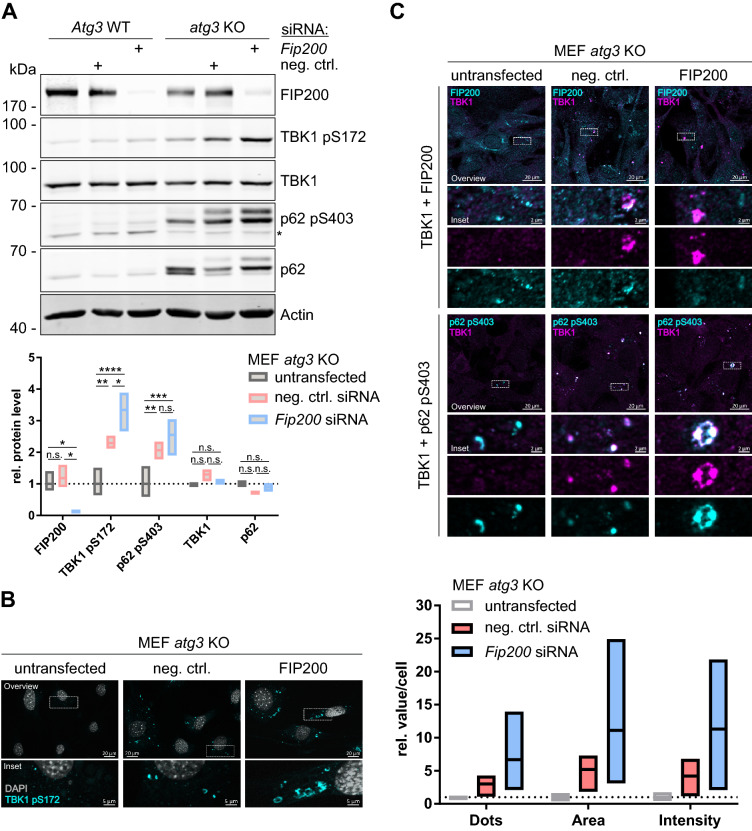
Loss of FIP200 further enhances TBK1 accumulation and activation caused by defective autophagy. (**A**) *Atg3* WT and *atg3* KO MEFs were transfected with either 20 nM negative control siRNA or 20 nM *Fip200* siRNA for 72 h, and the cleared cellular lysates were immunoblotted for the indicated proteins. The asterisk indicates a nonspecific band. The full-length blots are presented in Figure S9. The densities of the bands on immunoblots from three independent experiments were quantified and normalized to those of Actin. For each protein, the normalized densities of *atg3* KO samples were normalized to that of the respective untransfected sample. The boxes represent the highest and the lowest value, while the centerline shows the mean. Two-way ANOVA with Tukey's multiple comparisons test was used to determine differences between treatments. n.s. = not significant; *p < 0.05; **p < 0.01; ***p < 0.001; ****p < 0.0001. (**B + C**) *atg3* KO MEFs were transfected with either 20 nM negative control siRNA or 20 nM *Fip200* siRNA for 72 h and immunostained for TBK1 pS172 (B; fixed in 4% PFA) or TBK1 in combination with either FIP200 or SQSTM1/p62 pS403 (**C**; fixed in 100% MeOH). For (**B**), the number, area, and intensity of TBK1 pS172-positive structures of at least 232 cells per cell line were quantified using ImageJ 1.53c and normalized to those of untransfected *atg3* KO cells. The boxes represent the highest and the lowest value, while the centerline shows the mean from four independent experiments. For IF imaging, DAPI was used to stain nuclei.

Since we observed a clear difference between *atg3* KO and *fip200* KO MEFs with regard to phospho-TBK1 accumulation by IF (Fig. 2A) and a rather normal autophagic flux in FIP200 ΔCT cells (Fig. 5A), we speculated that inhibited autophagy could not be the sole cause for the aberrant TBK1 activation. Accordingly, we next investigated whether loss of FIP200 in autophagy-deficient cells can further increase TBK1 accumulation and activation. We performed FIP200 knockdown experiments in *atg3* KO cells and analyzed TBK1 activation and SQSTM1/p62 phosphorylation by immunoblotting. SiRNA-mediated knockdown of FIP200 led to increased TBK1 activation and SQSTM1/p62 phosphorylation in *atg3* KO MEFs (Fig. 6A). We also investigated TBK1 accumulation by IF. Upon transfection of nontargeting or *Fip200*-targeting siRNA, we observed punctate or circular TBK1-positive structures (Fig. 6B,C). *Fip200* siRNA led to the removal of FIP200 from these aggregates and to an increased size of the ring-shaped structures. Similar to the findings in *fip200* KO cells, the TBK1 structures also contained phosphorylated SQSTM1/p62 (Fig. 6C). Collectively, these data show that the combination of defective autophagy and loss of FIP200 leads to aberrant accumulation of TBK1-TAX1BP1-SQSTM1/p62 aggregates. It appears that the presence of FIP200 at these aggregates ensures the inducibility of TBK1 activation.

### The IFN-β response upon TLR3/TLR4 stimulation is not significantly affected in fip200 KO or FIP200 ΔCT-expressing cells

**Figure 7:**
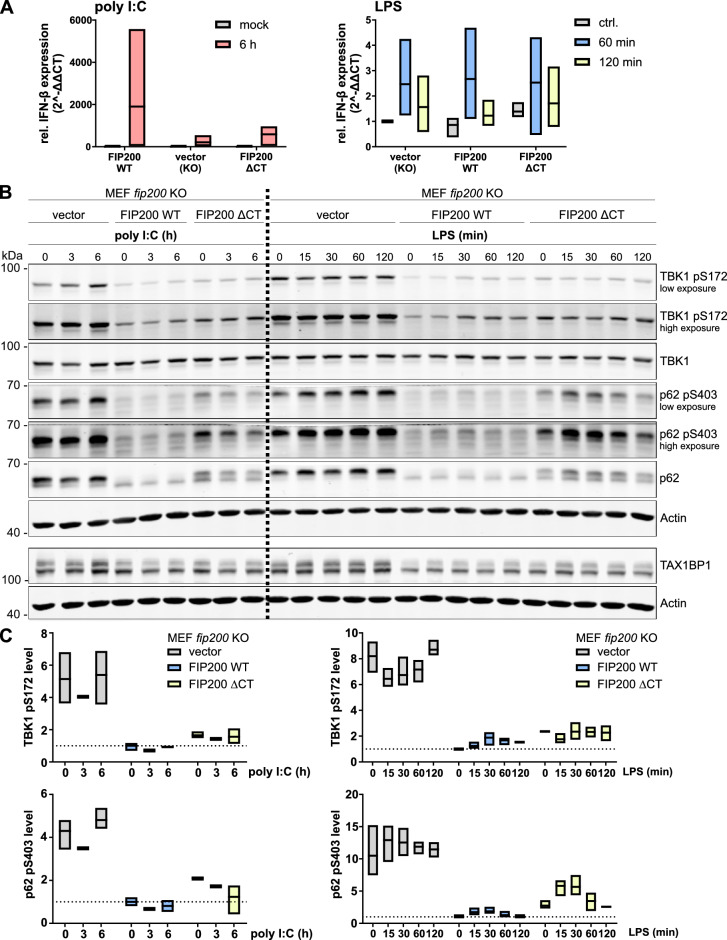
FIP200 deficiency desensitizes cells to TLR3-/TLR4-engaging stimuli. (**A–C**) *fip200* KO, FIP200 WT, and FIP200 ΔCT MEFs were transfected with 2 µg/ml poly I:C or treated with 1 µg/ml LPS for the indicated times. dH_2_O was used for mock transfection. (**A**) RT-qPCR analyses were performed to determine the relative IFN-β expression. All values were normalized to those of control samples of FIP200 WT MEFs. The boxes represent the highest and the lowest value, while the centerline shows the mean from three independent experiments. (**B**) Lysates of poly I:C-transfected or LPS-treated cells were immunoblotted for the indicated proteins. Representative immunoblots of three independent experiments are depicted. The full-length blots are presented in Figure S10. (**C**) The densities of the bands on immunoblots from three independent experiments (except for 3 h poly I:C transfection, which was performed only once) were quantified and normalized to those of Actin. The normalized densities of all samples were then normalized to that of the respective untreated sample of FIP200 WT cells. Only the relative levels of TBK1 pS172 and SQSTM1/p62 pS403 are shown. The boxes represent the highest and the lowest value, while the centerline shows the mean.

Since TBK1 is involved not only in autophagy signaling but also in the regulation of innate immune responses, we examined whether loss of FIP200 influences TLR3/TLR4-induced IFN-β production through TBK1-TAX1BP1-SQSTM1/p62 aggregate formation. *Fip200* KO, FIP200 WT and FIP200 ΔCT MEFs were either transfected with poly I:C or treated with lipopolysaccharide (LPS), and IFN-β gene expression was analyzed by reverse transcription (RT)-quantitative PCR (qPCR). In all three cell lines, poly I:C treatment resulted in clear upregulation of IFN-β mRNA expression, whereas the response to LPS was rather weak (Fig. 7A). Although TBK1 was highly activated in *fip200* KO MEFs, the activation did not result in increased IFN-β production under basal or stimulated conditions compared to that in FIP200 WT cells (Fig. 7A). In contrast, IFN-β production appeared rather impaired in *fip200* KO and FIP200 ΔCT MEFs after poly I:C treatment, but the differences were not statistically significant (Fig. 7A). Additionally, the receptors STING and TRIF, which recruit TBK1 during innate immune responses, did not colocalize with TBK1-TAX1BP1-SQSTM1/p62 aggregates (Figures S3A and S3B). In addition to IFN-β production, we also examined TBK1 and SQSTM1/p62 phosphorylation by immunoblotting. Neither poly I:C nor LPS induced major changes in the (increased) phosphorylation status of TBK1 and SQSTM1/p62 in *fip200* KO MEFs (Fig. 7B,C). Expression of wild-type FIP200 slightly restored the inducibility of TBK1 phosphorylation upon LPS treatment. In FIP200 ΔCT MEFs, poly I:C and LPS treatment also resulted in slightly increased TBK1 phosphorylation accompanied by a simultaneous decrease in SQSTM1/p62 phosphorylation (Fig. 7B,C). Taken together, the results suggest that FIP200 deficiency desensitizes cells to TLR3-/TLR4-engaging stimuli with regard to TBK1 activation. However, this desensitization does not result in increased IFN-β production under basal or TLR3-/TLR4-stimulated conditions. If FIP200 is present but not capable of interacting with TAX1BP1 and/or SQSTM1/p62 (as in FIP200 ΔCT MEFs), the inducibility of TBK1 activation upon TLR3/TLR4 engagement is partially restored.

## Discussion

As a component of the autophagy-inducing ULK1 complex, FIP200 is centrally involved in bulk and selective autophagy. However, FIP200 also participates in several additional cellular signaling pathways^38^. TBK1 is a central regulator of both autophagic and inflammatory signaling^39^. Here, we observed that TBK1, TAX1BP1 and SQSTM1/p62 form protein aggregates in MEFs lacking FIP200. Within these structures, TBK1 is activated by trans-autophosphorylation and in turn phosphorylates SQSTM1/p62 at Ser403, thus further promoting the formation of SQSTM1/p62-ubiquitin condensates. TBK1 activation and SQSTM1/p62 phosphorylation depend on TAX1BP1, indicating that this autophagy receptor mediates the recruitment of TBK1 to SQSTM1/p62 condensates. We observed that TBK1 activation is strongly enhanced in cells lacking FIP200. On the one hand, this might be caused by the inhibition of autophagy and thus the generally inhibited/reduced clearance of SQSTM1/p62 condensates. On the other hand, our data suggest that the constitutive binding of FIP200 to TBK1 via TAX1BP1 keeps TBK1 in an inducible state. Accordingly, FIP200 controls the TBK1 activation threshold at SQSTM1/p62-positive protein aggregates. Given this collective evidence, we hypothesize that the TAX1BP1-TBK1-phospho-SQSTM1/p62 axis engages in positive feedforward regulation of aggrephagy that is controlled by the recruitment of FIP200 (scheme depicted in Figure S4).

In 2008, it was reported that FIP200 binds to ULK1/2 and is required for autophagosome formation^35^. Since then, several reports have confirmed the central role of FIP200 in autophagy signaling, including in selective autophagic processes. Studies using conditional *fip200* KO mice have shown that loss of FIP200 leads to accumulation of SQSTM1/p62-positive protein aggregates in osteoblasts^40^, retinal pigment epithelial cells^41^, and mammary tumor cells^42^ due to impairment of autophagy. In addition, recent reports have indicated that recruitment of the ULK1 complex to damaged mitochondria or invading bacteria is mediated by the binding of FIP200 to the autophagy receptor NDP52^7,8^. Furthermore, it has been found that the C-terminal claw domain of FIP200 binds to the autophagy receptor SQSTM1/p62 and thus promotes autophagosome formation at SQSTM1/p62-ubiquitin condensates^33^. We observed that FIP200 also associates with the autophagy receptor TAX1BP1, similarly resulting in the association of FIP200 with SQSTM1/p62-positive condensates. Furthermore, it appears that NDP52 and/or OPTN is not recruited to these structures. Generally, our observations are in line with the findings of a recent study indicating that the clearance of protein aggregates is mediated by TAX1BP1^43^. Apparently, both SQSTM1/p62 and TAX1BP1 are present in protein aggregates and can recruit FIP200. The binding of FIP200 to these two autophagy receptors is not necessarily mutually exclusive, since binding of TAX1BP1 (and NDP52) has been reported to occur outside the claw domain^8^. However, TAX1BP1 is clearly responsible for TBK1 activation and SQSTM1/p62 phosphorylation within these structures.

Several types of crosstalk exist between TBK1 and autophagy. On the one hand, TBK1 regulates several components of the autophagy signaling cascade, including AMPK^10^, syntaxin 17^11^, and various autophagy receptors^7,8,12–17^. We observed that the TBK1-dependent phosphorylation of SQSTM1/p62 at Ser403 was increased in *fip200* KO MEFs. Such phosphorylation has been implicated in the SQSTM1/p62-dependent phase separation/clustering of polyubiquitinated proteins and the selective autophagic clearance of these protein aggregates^18,44,45^. Furthermore, it has been confirmed that TBK1 can catalyze this phosphorylation^13,14^. We propose that the TAX1BP1-dependent recruitment of TBK1 to protein aggregates and the subsequent phosphorylation of SQSTM1/p62 at Ser403 represent a feedforward loop that ensures efficient phase separation, engulfment, and clearance of the cargo. Apparently, this function needs to be controlled by FIP200, since TBK1 might increase the size and potentially the insolubility of the protein aggregates via uncontrolled SQSTM1/p62 phosphorylation. This conclusion is in line with the observations of Turco et al., who reported that knockdown of FIP200 increases both the number and volume of SQSTM1/p62 puncta^33^. As mentioned above, SQSTM1/p62-positive protein aggregates have already been reported for various cell lines obtained from conditional *fip200* KO mice^40–42^. Additionally, TBK1 inhibition has been found to prevent the generation of insoluble protein aggregates in hepatocytes^46^. Interestingly, we observed that the phosphorylated-Ser403 (pS403) variant of SQSTM1/p62 shifted to an increased molecular weight. Ser403 phosphorylation itself might have contributed to this phenomenon; however, we cannot exclude the possibility that additional phosphorylation events were involved. Turco et al. reported that phosphorylation of the FIP200-interacting region in SQSTM1/p62 (i.e., Ser349/Thr350, Ser365, Ser366, Ser370/Thr375) increases the affinity of SQSTM1/p62 for the FIP200 claw domain^47^. It is possible that phosphorylation of this region increases as a compensatory mechanism to counteract FIP200 deficiency and thus contributes to the alteration in molecular weight.

On the other hand, TBK1 activation is controlled by autophagy, as confirmed by our observations. We found that inhibition of autophagy via targeting of various autophagy-related genes (e.g., ATG3, FIP200, ATG13, ULK1) or via treatment with BafA_1_ increased TBK1 activation. Furthermore, starvation-induced autophagy decreased TBK1 activation, but starvation did not affect TBK1 activation in autophagy-incompetent cells. Yang et al. also observed that starvation-induced autophagy represses TBK1 activation, while inhibition of autophagy increases TBK1 activation^48^. The authors suggested that NDP52 and SQSTM1/p62 promote autophagy of phospho-TBK1 complexes^48^. It has also been reported that TBK1 is directly targeted by ULK1^10^. Goodwin et al. analyzed TBK1 activation during lysosomal ferritin flux in cells deficient in either of the ULK1 complex subunits. Similar to our results, their findings revealed that among the alterations, FIP200 depletion had the greatest impact on TBK1 activation; the authors also observed TAX1BP1-TBK1-positive structures^34^. In that case, the authors suggested that a compensatory relationship exists between the ULK1/2 complex and TBK1 activation, at least for lysosomal ferritin flux^34^. We propose that FIP200 exerts an additional specific function during aggrephagy/selective autophagy that cannot be compensated for by the other subunits of the ULK1 complex. Furthermore, we propose that this additional function of FIP200 is independent of its autophagy-regulating function. These propositions are supported by two observations. First, we detected increased phospho-TBK1 levels in cells deficient in ULK1/2, ATG13, or ATG3. However, the levels in these cells were still lower than those in *fip200* KO MEFs. We believe that the number and/or size of protein aggregates was increased in all these cell lines with autophagy deficiency/impairment and that this effect certainly contributed to TBK1 activation. However, these data simultaneously suggest that the recruitment of the ULK1 complex is not the sole function of FIP200 during aggrephagy. Of note, Turco et al. reported that ULK1 is still recruited to SQSTM1/p62 condensates in cells lacking FIP200 but that ATG16L1 recruitment is abolished^33^. This observation was recently substantiated in a preprint from the same group reporting that FIP200 is dispensable for the recruitment of the upstream autophagy machinery to the condensates but necessary for phosphatidylinositol 3-phosphate formation, WIPI2 recruitment, and ULK1 kinase activation^49^. Second, we observed increased phospho-TBK1 levels in cells expressing the ΔCT variant of FIP200. However, in these cells, normal starvation-induced autophagy occurred. It has been reported that the ULK1 complex is organized by a C-shaped FIP200 N-terminal domain dimer^50^. Accordingly, it is possible that the C-terminal domain of FIP200 exerts some additional functions that are independent of its canonical autophagy function. Notably, Jun-Lin Guan’s group recently reported that FIP200 can limit AZI2/NAP1-TBK1-IRF signaling independent of its canonical autophagy function^51,52^. They proposed that—in *fip200* KO cells—phospho-TBK1-containing phase condensates accumulate, resulting in TBK1 hyperactivation and sustained proinflammatory signaling^51,52^. This mechanism is in line with a report describing that cellular stress leads to incorporation of the TBK1 adaptors TBKBP1/SINTBAD and AZI2/NAP1 into membraneless organelles that control the threshold of TBK1 activation^53^. Whether the TBKBP1/SINTBAD-AZI2/NAP1-membraneless organelles and the SQSTM1/p62 aggregates represent different or overlapping phase condensates remains to be investigated. We detected TBKBP1/SINTBAD in our MS analyses of anti-FIP200 immunopurifications (see Table S1), but we did not observe differences in TBKBP1/SINTBAD puncta formation in FIP200 WT, FIP200 ΔCT or *fip200* KO cells (data not shown). It is tempting to speculate that different compositions of phase condensates can regulate different TBK1 signaling outcomes in different cellular systems. Here, we observed an effect not on IFN-β production but rather on SQSTM1/p62 phosphorylation. Generally, the relative contributions of the different protein–protein interactions for the heterotrimeric control of TBK1 activation at phase condensates, i.e., the interactions between FIP200 and TAX1BP1 (see this report and^8^), the interactions between TAX1BP1 and TBKBP1/SINTBAD or AZI2/NAP1^16^, and the interactions between FIP200 and TBKBP1/SINTBAD or AZI2/NAP1, await further clarification^8,51^. Furthermore, it has been suggested that TAX1BP1 can directly associate with TBK1^12^, adding another level of complexity. Currently, we speculate that the binding of FIP200 to TAX1BP1 and/or TBKBP1/SINTBAD-AZI2/NAP1 is sufficient for control of TBK1 activation either via mediation of the selective removal of TBK1 or via steric hindrance of TBK1 trans-autophosphorylation.

The involvement of LC3 in this process (removal of SQSTM1/p62 condensates and/or regulation of TBK1 activation) needs to be clarified. In the abovementioned study on TBK1 hyperactivation at phase condensates, the authors used a FIP200 variant that cannot bind ATG13^51^. In cells expressing this mutant, LC3 lipidation is blocked^54^. The authors speculated that FIP200 can—through its ability to bind cargo receptors—bypass the need for LC3 and initiate phagophore formation at the cargo during selective autophagy^52^. However, alternative recruitment mechanisms might also play roles. It has been reported that deletion of the FIP200 claw domain does not completely prevent the targeting of SQSTM1/p62-positive cargo to lysosomes^33^ and that there is residual LC3 lipidation in cells expressing a FIP200 claw domain mutant that cannot bind to SQSTM1/p62^49^. We clearly observed partial colocalization of LC3 and TBK1 in both *fip200* KO cells and cells expressing our ΔCT variant lacking both the interaction site with TAX1BP1 and the claw domain. At present, we cannot differentiate whether this colocalization was caused by alternative recruitment of the LC3 lipidation machinery or perhaps by lipidation-independent recruitment of LC3 molecules.

Protein aggregation is a hallmark of neurodegenerative diseases. Additionally, several mutations in *TBK1* have been identified that underlie neuroinflammatory diseases, and dysregulation of TBK1 is strongly connected to these diseases (reviewed in^30^). For example, treatment with the TBK1 inhibitor BX795 abrogates the aberrant insolubility of an optineurin mutant (E50K) that can be found in patients suffering from NTG^55^. Additionally, ALS-associated *TBK1* mutations affect the phosphorylation of different autophagy receptors^56^. A role of FIP200 in neuronal homeostasis has also been recognized. Liang et al. reported that neural-specific loss of FIP200 resulted in cerebellar degeneration accompanied by progressive neuronal loss, spongiosis, and neurite degeneration^57^. The authors also reported accumulation of SQSTM1/p62 and ubiquitinated protein aggregates^57^. Interestingly, the observed phenotypes developed earlier and were somewhat more severe in neural-specific FIP200 conditional KO mice than in Atg5 or Atg7 conditional KO mice^57^. We speculate that the FIP200-dependent regulation of TBK1 activity at SQSTM1/p62 condensates might contribute to these observations, establishing enforced recruitment of FIP200 to these condensates—next to pharmacological regulation of TBK1 activity—as a promising therapeutic approach.

## Materials and methods

### Antibodies and reagents

Antibodies against β-Actin (western blot [WB]: 1:20,000, clone AC-74, Sigma-Aldrich, #A5316), ATG13 (1:1000, Sigma-Aldrich, #SAB4200100), ATG3 (WB: 1:1000, Cell Signaling Technology, #3415), ERGIC-53/p58 (IF: 1:25, Sigma-Aldrich, #E1031), ERp72 (IF: 1:100, clone D70D12, Cell Signaling Technology, #5033), FIP200 ([1] IF: 1:500, Proteintech, #17250-1-AP, [2] WB: 1:1000, Bethyl Laboratories, #A301-536A or [3] WB: 1:1000, Bethyl Laboratories, #A301-574A, used for THP-1 and HeLa cells), FLAG (WB: 1:1000, clone M2, Sigma-Aldrich, #F1804), GAPDH (WB: 1:5000, clone 6C5, Abcam, #ab8245), GFP (WB: 1:1000, clone 3H9, ChromoTek, #3H9), Golgin97 (IF: 1:100, clone D8P2K, Cell Signaling Technology, #13192), GST (IF: 1:800, clone 26H1, Cell Signaling Technology, #2624), HA (WB: 1:2000, BioLegend, #901501), LC3B (WB: 1:1000, IF: 1:200, Cell Signaling Technology, #2775), NDP52 (IF: 1:50, Proteintech, #12229-1-AP), OPTN (IF: 1:50, Proteintech, #10837-1-AP), SQSTM1/p62 (WB: 1:1000, PROGEN, #GP62-C), SQSTM1/p62 pS403 (WB: 1:1000, IF: 1:400, clone D8D6T, Cell Signaling Technology, #39786), STING (IF: 1:100, Proteintech, #19851-1-AP), TAX1BP1 ([1] IF: 1:50, Proteintech, #14424-1-AP or [2] WB: 1:1000, Bethyl Laboratories, #A303-792A), TBK1 ([1] IF: 1:50, WB: 1:1000, clone A-6, Santa Cruz Biotechnology, #sc-398366 or [2] WB: 1:1000, clone D1B4, Cell Signaling Technology, #3504, used for THP-1 and HeLa cells), TBK1 pS172 (IF: 1:50, WB: 1:1000, clone D52C2, Cell Signaling Technology, #5483), TRIF (IF: 1:100, clone E-7, Santa Cruz Biotechnology, #sc-514384), ULK1 (1:1000, clone D8H5, Cell Signaling Technology, #8054), and Vinculin (WB: 1:2000, clone hVIN-1, Sigma-Aldrich, #V9131) were used. For immunoblot analyses, IRDye 800- or IRDye 680-conjugated secondary antibodies were used (LI-COR Biosciences, #926-68070 [680RD goat anti-mouse IgG], #926-68071 [680RD goat anti-rabbit IgG], #926-32211 [800CW goat anti-rabbit IgG], #926-32219 [800CW goat anti-rat IgG], and #926-68077 [680RD donkey anti-guinea pig IgG]). The secondary antibodies for IF analyses were purchased from Jackson ImmunoResearch (Alexa Fluor 488-AffiniPure goat anti-rabbit IgG, 1:500, #111-545-003 and Alexa Fluor 647-AffiniPure goat anti-mouse IgG, 1:500, #115-605-003). Other reagents used were BafA_1_ (Sigma-Aldrich, #B1793), LPS from *Escherichia coli* O55:B5 (Sigma-Aldrich, #L4524), PMA (Sigma-Aldrich, #P1585), polyinosinic:polycytidylic acid (poly I:C; Sigma-Aldrich, #P9582), and puromycin (InvivoGen, #ant-pr). Dimethyl sulfoxide (DMSO; AppliChem, #A3672) was used to dissolve BafA_1_.

### Constructs and siRNAs

Human cDNA encoding FLAG-tagged full-length FIP200 (isoform 1) was amplified from p3xFLAG-CMV10-hFIP200 (kindly provided by Noboru Mizushima and previously described in^35^; Addgene plasmid #24300; http://n2t.net/addgene:24300; RRID: Addgene_24300) and cloned into the multiple cloning site of the oncoretroviral vector S91I2Pco. This vector expressed *FIP200* cDNA and an internal ribosomal entry site (IRES)-puromycin resistance gene (*paca*) cassette optimized for human codon usage using the GeneArt online tool (Thermo Fisher Scientific) under the control of the SFFV promoter in the 5'LTR. The codons encoding the C terminus of FIP200 (amino acids 1369–1594, CT) were removed by site-directed mutagenesis using Q5 High-Fidelity 2X Master Mix (New England Biolabs, #M0492). The sequences of oligonucleotides used for mutagenesis PCR were TAAAATTCTGCAGTCGAC (FIP200 Δ1369-1594 fwd) and TATCAAATCTTTATCCCGTTC (FIP200 Δ1369-1594 rev). The FIP200-encoding S91I2Pco vectors (both full-length and ΔCT) harbored cDNAs encoding the amino acid exchange D818Y. Murine *Ulk1* and *Ulk2* cDNAs were kindly provided by Do-Hyung Kim (Addgene plasmid #31960; http://n2t.net/addgene:31960; RRID: Addgene_31960) and Masaaki Muramatsu (Addgene plasmid #22898; http://n2t.net/addgene:22898; RRID: Addgene_22898), respectively. pMSCVblast-MYC-mULK1 was generated by amplifying *MYC-Ulk1* using the oligonucleotides 5´ gattaactcgagATGGAGCAAAAGCTCATTTCTGAGG 3´ (forward) and 5´ ctagttgttaacTCAGGCATAGACACCACTCAGC 3´ (reverse), digestion with *XhoI* and *HpaI* (Thermo Scientific #FD0694 and #FD1034) and cloning into pMSCVblast digested with the same enzymes. Subsequently, pMSCVblast-3xFLAG-mULK1 was generated by replacing the codons encoding the MYC tag in the pMSCVblast-MYC-mULK1 vector with codons encoding 3xFLAG by site-directed mutagenesis using the oligonucleotides 5´ gattataaagatcatgacatcgattacaaggatgacgatgacaagGGATTCCTGGAGCCGGGCCGCGGCGGCGTCGAG 3´ (forward) and 5´ accgtcatggtctttgtagtccatCGTTAACCTCGAGAGATCTAATTCCGGCGCCTAGAGAAGGAGTGAGGG 3´ (reverse). After amplification, the PCR product was treated with KDL enzyme mix (NEB # M0554S) and transformed into competent cells. pMSCVzeo-3xHA-mULK2 was generated by amplification of the 3xHA-mULK2 fragment with the oligonucleotides 5´gcgccggaattagatctctcgaggttaacgATGGCATACCCTTATGACGTAC 3´ (forward) and 5´ ggaaaagcgcctcccctacccggtagaattTCACACAGTTGCAGTGCTACAG 3´ (reverse) and insertion into the pMSCVzeo plasmid (a gift from David Mu; Addgene plasmid #75088; http://n2t.net/addgene:75088; RRID: Addgene_75088). For this, pMSCVzeo was linearized with *EcoRI* (Thermo Fisher Scientific #FD0274), and both the 3xHA-mULK2 PCR product and the linearized pMSCVzeo were treated with 10 U of Klenow fragment (Thermo Fisher Scientifc #EP0054), purified and annealed in the presence of ligase buffer (Thermo Fisher Scientific #B69). The reaction was incubated for 30 min at 37 °C and used to transform competent cells. Mouse *Fip200* (ON-TARGETplus siRNA SMARTpool, #L-041191-01-0005), mouse *Tax1bp1* (ON-TARGETplus siRNA SMARTpool, #L-055360-01-0005) and negative control (ON-TARGETplus nontargeting pool, #D-001810-10-20) siRNAs were obtained from Dharmacon (Horizon Discovery Group). Human *FIP200* (#HSS114818 [#1], #HSS114819 [#2] and #HSS190643 [#3]), human *GAPDH* (#12935-140, RNAi Positive Control) and negative control (#12935-300, Medium GC Duplex) Stealth siRNAs were obtained from Thermo Fisher Scientific. Lipofectamine RNAiMAX (Thermo Fisher Scientific, #13778-150) was used as a transfection reagent to knock down FIP200 and TAX1BP1 in MEFs, and Viromer Blue (Biozym Scientific, #230005) was used to knock down FIP200 and GAPDH in HeLa and THP-1 cells. Seventy-two hours after transfection, cells were harvested for immunoblotting or fixed and stained for IF.

### Cell lines

*Fip200* KO MEFs were kindly provided by Jun-Lin Guan (Department of Cancer Biology, University of Cincinnati College of Medicine, Cincinnati, Ohio, USA) and have been described previously^58^. *Ulk1/2* double knockout (DKO) MEFs were kindly provided by Tullia Lindsten (Memorial Sloan Kettering Cancer Center, New York City, New York, USA) and have been described previously^59^. Plat-E cells were kindly provided by Toshio Kitamura (Institute of Medical Science, University of Tokyo, Japan) and have been described previously^60^. The Plat-E cells were transfected with 1.9 µg of the S91I2Pco- or pMSCV-based retroviral vectors using FuGENE 6 Transfection Reagent (Promega, #E2692) to produce recombinant retroviruses. After 48 h, retroviral supernatant was collected and added together with 3 µg/ml polybrene (Sigma, #9268) to *fip200* KO or *ulk1/2* DKO MEFs. After 72 h, cells were selected in medium containing puromycin (2.5 µg/ml; InvivoGen #ant-pr-1), blasticidin (35 µg/ml; InvivoGen #ant-bl-05), or zeocin (Invitrogen #46-0509). *Atg13* KO MEFs were kindly provided by Noboru Mizushima (Department of Biochemistry and Molecular Biology, Graduate School and Faculty of Medicine, University of Tokyo, Japan), and their reconstitution has been described previously^61^. Wild-type and *atg3* KO MEFs were kindly provided by Masaaki Komatsu (Department of Physiology, Juntendo University Graduate School of Medicine, Tokyo, Japan) and have been described previously^62^. Wild-type HeLa cells were kindly provided by Richard J. Youle (National Institute of Neurological Disorders and Stroke, Bethesda, Maryland, USA). THP-1 cells were obtained from the Leibniz Institute DSMZ-German Collection of Microorganisms and Cell Cultures (#ACC 16). The procedure for generating Flp-In T-REx 293 cells inducibly expressing a protein of interest has been previously described^63^. Briefly, full-length human FIP200 cDNA was amplified and cloned into the vector pcDNA5/FRT/TO-GFP. The expression constructs (GFP or GFP-FIP200) were then cotransfected with pOG44 into Flp-In T-REx 293 cells (Invitrogen, R780-07). Stable transfectants were selected with 200 μg/ml hygromycin B (Invitrogen, 10687-010) and 5 μg/ml blasticidin (Invitrogen, A11139-02). MEFs, HeLa cells and Flp-In T-REx 293 cells were cultured in high-D-glucose Dulbecco’s modified Eagle’s medium (DMEM, Thermo Fisher Scientific, #41965-039) supplemented with 10% FCS (GE Healthcare, #A15-101 or Thermo Fisher Scientific, #10270-106), 100 U/ml penicillin and 100 μg/ml streptomycin at 37 °C and 5% CO_2_. THP-1 cells were cultured in Roswell Park Memorial Institute (RPMI) 1640 medium (Thermo Fisher Scientific, #61870-010) supplemented with 10% FCS, 100 U/ml penicillin, 100 μg/ml streptomycin and 10 mM HEPES solution (Sigma-Aldrich, #H0887). For amino acid starvation, cells were washed once with PBS and starved in EBSS (Thermo Fisher Scientific, #24010043) for 2 h. For induction of GFP or GFP-FIP200 expression, Flp-In T-REx 293 cells were incubated in complete medium containing 0.1 doxycycline (Clontech Laboratories, #631311) for 16 h.

### IF

For IF microscopy, MEFs were seeded on glass coverslips (Marienfeld). For staining of total TBK1, cells were fixed in cold methanol for 15 min at 4 °C. Whenever total TBK1 was not stained, cells were fixed in 4% paraformaldehyde (PFA) for 15 min at 4 °C, quenched with 50 mM NH_4_Cl for 15 min and permeabilized with 50 µg/ml digitonin (Sigma-Aldrich, #D141) for 5 min. The fixed samples were blocked with 3% BSA (Roth, #8076) for 30 min and incubated with primary antibodies diluted in 3% BSA for 1–2 h. Afterwards, the samples were washed three times with PBS, incubated with secondary antibodies diluted in 3% BSA for 30 min, and washed once with 0.2% Tween-20 (Sigma-Aldrich, #P1379) and three times with PBS. Finally, the cells were embedded in ProLong Glass Antifade Mountant (Thermo Fisher Scientific, #P36980) containing 1 µg/ml DAPI (Roth, #6335.1). Representative images were collected with an Axio Observer 7 fluorescence microscope (Carl Zeiss Microscopy) using a 40x/1,4 Oil DIC M27 Plan-Apochromat objective (Carl Zeiss Microscopy) and an ApoTome 2 (Carl Zeiss Microscopy) using the 488 nm and 647 nm channels. A lateral shift in channel 647 was corrected using ZEN 2.3 (Carl Zeiss Microscopy) as follows: x = −0.82 px (63.5 nm) and y = 0.52 px (40.3 nm). The average displacement values were estimated from five images of multispectral beads (FocalCheck fluorescence microscope test slide #1 A5, Invitrogen, # F36909), and the analysis was based on the channel registration tool of the NanoJ toolbox for ImageJ using a single block to obtain linear displacement values^64^. The pixel intensities of the areas indicated by dashed red arrows were measured with ZEN 2.3 lite (Carl Zeiss Microscopy) and are depicted in bar graphs.

### Immunoblotting and immunopurification

For immunoblotting, cells were harvested following the indicated treatment, pelletized, flash-frozen and lysed in lysis buffer (50 mM Tris–HCl [pH 7.5], 150 mM NaCl, 1 mM EDTA, 1 mM EGTA, 10 μM Na_2_MoO_4_, 1 mM Na_3_VO_4_, 50 mM NaF, 5 mM Na_4_P_2_O_7_, 1% [v/v] Triton X-100 [Carl Roth, #3051.2], and protease inhibitor cocktail [Sigma-Aldrich, #P2714]) for 30 min on ice. The lysates were clarified by centrifugation at 18 000 rcf and 4 °C for 15 min. The protein concentrations were determined by the Bradford method, and the samples were adjusted to equal concentrations. The samples were then prepared via addition of sample buffer (125 mM Tris–HCl [pH 6.8], 17.2% [v/v] glycerol, 4.1% [w/v] SDS [AppliChem GmbH, #A7249], 200 μg/ml bromophenol blue, 2% [v/v] β-mercaptoethanol), heated to 95 °C for 5 min and subjected to SDS-PAGE. Afterwards, the proteins were transferred to PVDF membranes (Merck Millipore, #IPFL00010) and analyzed using the indicated primary antibodies and appropriate secondary antibodies (LI-COR Biosciences). The signals were detected using an Odyssey Infrared Imaging system (LI-COR Biosciences) and quantified with Image Studio 5.25 (LI-COR Biosciences). For affinity purification of GFP-tagged proteins, cells were flash-frozen and lysed in lysis buffer containing 0.3% CHAPS (Roth, #1479.2) as detergent. The lysates were clarified and incubated with GFP-trap beads (ChromoTek, #gta-200) overnight with rotation. The purified proteins were washed three times with lysis buffer and analyzed by immunoblotting.

### RT-qPCR

Total RNA was isolated from approximately 1 × 10^6^ cells using a NucleoSpin RNA II Kit (Macherey–Nagel, #740955.250) according to the manufacturer’s instructions. The isolated RNA was either directly used for reverse transcription or stored at − 80 °C. First-strand cDNA was generated using a High-Capacity cDNA Reverse Transcription Kit (Thermo Fisher Scientific, #4368814) according to the manufacturer’s instructions with 1 μg of isolated total RNA plus additional RNase inhibitor (Thermo Fisher Scientific, #10777019) and standard cycling conditions (10 min at 25 °C, 120 min at 37 °C, 5 min at 85 °C). The generated cDNA was directly used for qPCR or stored at − 20 °C. qPCR analysis was performed using an Applied Biosystems 7300 Real Time PCR System (Thermo Fisher Scientific) and GoTaq qPCR Master Mix (Promega, #A6001). The sequences of the oligonucleotides used for RT-qPCR were TGACAGGATGCAGAAGGAGA (β-Actin left), CGCTCAGGAGGAGCAATG (β-Actin right), CAGGCAACCTTTAAGCATCAG (IFN-β left), and CCTTTGACCTTTCAAATGCAG (IFN-β right). For each gene, technical triplicates were prepared, each of which contained 25 ng of total cDNA and a primer concentration of 500 nM. Amplification was performed with a standard temperature profile (2 min at 50 °C, 10 min at 95 °C, and 40 cycles of 15 s at 95 °C and 1 min 60 °C) followed by a dissociation run (15 s at 95 °C, 1 min at 60 °C, gradual increase of 1 °C/min up to 95 °C). The cycle threshold (CT) values were computed automatically. Relative gene expression was calculated by the 2-∆∆CT method, and β-Actin was used as a reference gene. Additionally, the values were normalized to those of control samples. The experiments were performed in triplicate.

### In-gel digestion and MS

For MS analysis, gel pieces were reduced, alkylated and digested with trypsin as described elsewhere^65^. MS was performed as previously described^66^: Peptides were extracted with 0.1% trifluoroacetic acid and subjected to liquid chromatography (LC). For peptide separation over a 130 min LC gradient, an Ultimate 3000 Rapid Separation LC system (Dionex/Thermo Fisher Scientific, Idstein, Germany) equipped with an Acclaim PepMap 100 C18 column (75 μm inner diameter, 25 cm length, 2 µm particle size from Thermo Fisher Scientific, Bremen, Germany) was used. MS analysis was carried out on an Orbitrap Elite mass spectrometer (Thermo Scientific, Bremen, Germany) operating in positive mode and equipped with a nano-electrospray ionization source. The capillary temperature was set to 275 °C, and the source voltage was set to 1.4 kV. Survey scans were carried out in the Orbitrap mass analyzer over a mass range from 350 to 1700 m/z at a resolution of 60 000 (at 400 m/z). The target value for the automatic gain control was 1 000 000, and the maximum fill time was 200 ms. The 20 most intense peptide ions (minimum signal intensity 500, excluding singly charged ions and ions with a charge state ≥ 4) were isolated, transferred to the linear ion trap (linear trap quadrupole, LTQ) part of the instrument and fragmented using collision-induced dissociation. The peptide fragments were analyzed using a maximum fill time of 200 ms and an automatic gain control target value of 100 000 with a mass range set depending on the parent mass using normal scan mode. Already fragmented ions were excluded from fragmentation for 45 s.

### Computational MS data analysis

The computational analysis of the MS data was based on previously described protocols^67^. Peptide and protein identification and quantification were performed using MaxQuant (version 1.5.0.3, MPI for Biochemistry, Planegg, Germany) applying standard parameters. Searches were carried out based on 20183 *Homo sapiens* protein entries downloaded from UniProtKB on 26th November 2014. Methionine oxidation and acetylation at the protein N termini were set as variable modifications, and carbamidomethylation at cysteines was considered as fixed modification. Peptides and proteins were accepted with a false discovery rate set to 1%. Unique and razor peptides were used for label-free quantification. The minimal ratio count was set to two, and the “match between runs” option was enabled. The normalized intensities provided by MaxQuant were analyzed using the Perseus framework (version 1.5.0.15, MPI for Biochemistry, Planegg, Germany). Only proteins with a minimum of 3 valid values in total were considered for protein quantification. Proteins that were identified only by site or were marked as contaminants (from the MaxQuant contaminant list) were excluded from the analysis. For calculation of enriched proteins in the two groups, Student’s t-test was applied. The significance analysis was applied to log2-transformed values after replacement of missing values from a normal distribution (width 0.3, downshift 1.5) using an S0 constant = 0 and a 5% false discovery rate-based cutoff^68^. The MS proteomics data have been deposited to the ProteomeXchange Consortium via the PRIDE partner repository with the data set identifier PXD022563.

### Statistical analysis

For immunoblotting, the density of each protein band was divided by the average density of all bands from the same protein on the membrane. The resulting ratios for the proteins of interest were normalized to the ratios of the corresponding loading controls, and the fold changes were calculated by dividing each normalized density ratio by the average density ratio of the indicated control lane (control lane: fold change = 1.00, n ≥ 3). For IF analyses, puncta and nuclei were quantified and analyzed using ImageJ 1.53c. A punctum-to-nucleus ratio was calculated to determine the number of puncta per cell. The macros used for quantifications are provided in Table S2. At least 50 cells per experiment were analyzed in at least three biological replicates. For comparisons between different groups, two-way ANOVA was performed using GraphPad Prism 7 (GraphPad Software).

## Supplementary Information

Supplementary Information 1.

Supplementary Information 2.

Supplementary Information 3.

## Data Availability

Further information and requests for resources and reagents should be directed to and will be fulfilled by the corresponding author. The plasmids generated in this study are available from the corresponding author without restriction upon request. The mass spectrometry proteomics data have been deposited to the ProteomeXchange Consortium^69^ via the PRIDE^70^ partner repository with the dataset identifier PXD022563.

## Abbreviations

ALS: Amyotrophic lateral sclerosis
ATG: Autophagy-related
AZI2/NAP1: 5-Azacytidine-induced 2
cGAS: Cyclic GMP-AMP synthase
FIP200: Focal adhesion kinase (FAK)-interacting protein of 200 kDa
FTD: Frontotemporal dementia
HSE: Herpes simplex encephalitis
IFN: Interferon
IRF3/7: Interferon regulatory factor 3/7
(MAP1) LC3: (Microtubule-associated protein 1A/1B) light chain 3
MAVS: Mitochondrial antiviral signaling protein
MyD88: Myeloid differentiation primary response 88
NBR1: Neighbor of BRCA1 gene 1
NDP52: Nuclear dot protein 52 kDa
NRBF2: Nuclear receptor-binding factor 2
NTG: Normal-tension glaucoma
OPTN: Optineurin
PIK3C3/VPS34: Phosphatidylinositol 3-kinase catalytic subunit type 3
PIK3R4/VPS15: Phosphoinositide 3-kinase regulatory subunit 4
PRR: Pattern recognition receptor
RIG-I: Retinoic acid-inducible gene I
RLR: RIG-I-like receptor
SAR: Selective autophagy receptor
SQSTM1/p62: Sequestosome 1
STING: Stimulator of interferon genes
TAX1BP1: Tax1-binding protein 1
TBK1: TANK-binding kinase 1
TBKBP1/SINTBAD: TBK1-binding protein 1
TLR3/4: Toll-like receptor 3/4
TRIF: TIR-domain-containing adapter-inducing interferon-β
ULK1/2: UNC-51-like autophagy-activating kinase 1/2
