# Supplementary material for: FIP200 controls the TBK1 activation threshold at SQSTM1/p62-positive condensates
Source: Sci Rep. 2021 Jul 5;11:13863. doi: 10.1038/s41598-021-92408-4 (PMC8257712; doi:10.1038/s41598-021-92408-4)
Supplement: Supplementary file 1 — Supplementary Information 1. [file 41598_2021_92408_MOESM1_ESM.docx]

FIP200 controls the TBK1 activation threshold at SQSTM1/p62-positive condensates

David Schlütermann^1^, Niklas Berleth^1^, Jana Deitersen^1^, Nora Wallot-Hieke^1^, Olena Friesen^1^, Wenxian Wu^1^, Fabian Stuhldreier^1^, Yadong Sun^1^, Lena Berning^1^, Annabelle Friedrich^1^, María José Mendiburo^1^, Christoph Peter^1^, Constanze Wiek^2^, Helmut Hanenberg^2,3^, Anja Stefanski^4^, Kai Stühler^1,4^, Björn Stork^1,*^

^1^*Institute of Molecular Medicine I, Medical Faculty and University Hospital Düsseldorf, Heinrich Heine University Düsseldorf, 40225 Düsseldorf, Germany*

^2^*Department of Otorhinolaryngology & Head/Neck Surgery, Medical Faculty and University Hospital Düsseldorf, Heinrich Heine University Düsseldorf, 40225 Düsseldorf, Germany*

^3^*Department of Pediatrics III, University Hospital Essen, University of Duisburg-Essen, 45122 Essen, Germany*

^4^*Molecular Proteomics Laboratory, Biologisch-Medizinisches Forschungszentrum (BMFZ), Heinrich Heine University Düsseldorf, 40225 Düsseldorf, Germany*

**Supplementary Figures S1-S12**

**Legends to Supplementary Tables S1 and S2**

**
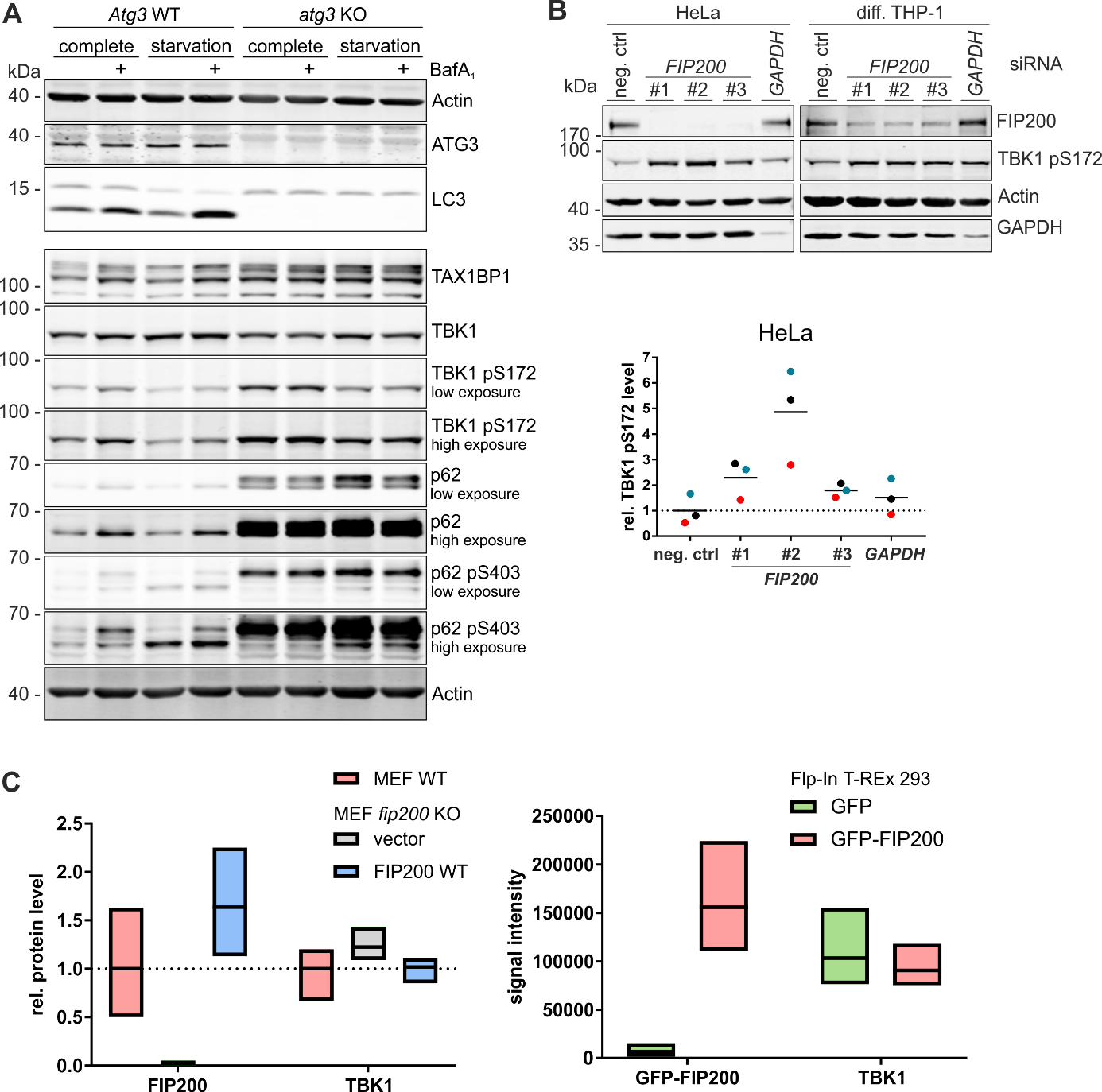
**

**Supplementary Figure S1:** **TBK1 activation is increased in *atg3* KO MEFs and in human cells treated with *FIP200* siRNA, but FIP200 overexpression does not affect TBK1 protein levels.** (**A**) *Atg3* WT and *atg3* KO MEFs were cultured in complete or starvation (EBSS) medium in either the absence or presence of 10 nM BafA_1_ for 2 h. Cleared cellular lysates were immunoblotted for the indicated proteins. The full-length blots are presented in Figure **S11**. (**B**) THP-1 cells were incubated with 100 ng/ml PMA for 72 h to induce differentiation. HeLa and differentiated THP-1 cells were transfected with 20 nM negative control siRNA, 20 nM *FIP200* siRNA (#1, #2 or #3), or 20 nM *GAPDH* siRNA for 72 h. Cleared cellular lysates were immunoblotted for the indicated proteins. For HeLa cells, the densities of the bands on immunoblots from three independent experiments were quantified and normalized to those of Actin. All values were then normalized to those of the samples transfected with the negative control siRNA. The dots represent the relative TBK1 pS172 levels in each experiment, and each color indicates one experiment. The lines show the mean from the three independent experiments. The full-length blots are presented in Figure **S12**. (**C**) For WT MEFs (MEF WT) and *fip200* KO MEFs transfected with empty vector or with cDNA encoding full-length FIP200 (FIP200 WT) (left diagram), the densities of the TBK1 and FIP200 bands on immunoblots from three independent experiments were quantified and normalized to those of Vinculin. The normalized densities of all samples of each protein were then normalized to those of the MEF WT samples. Only the relative levels of TBK1 and FIP200 are shown. For Flp-In T-REx 293 cells inducibly expressing GFP or GFP-FIP200 (right diagram), the densities of the TBK1 and GFP-FIP200 bands on immunoblots from three independent experiments were quantified, and the total signal intensities are shown. The boxes represent the highest and the lowest value, while the centerline shows the mean.

**
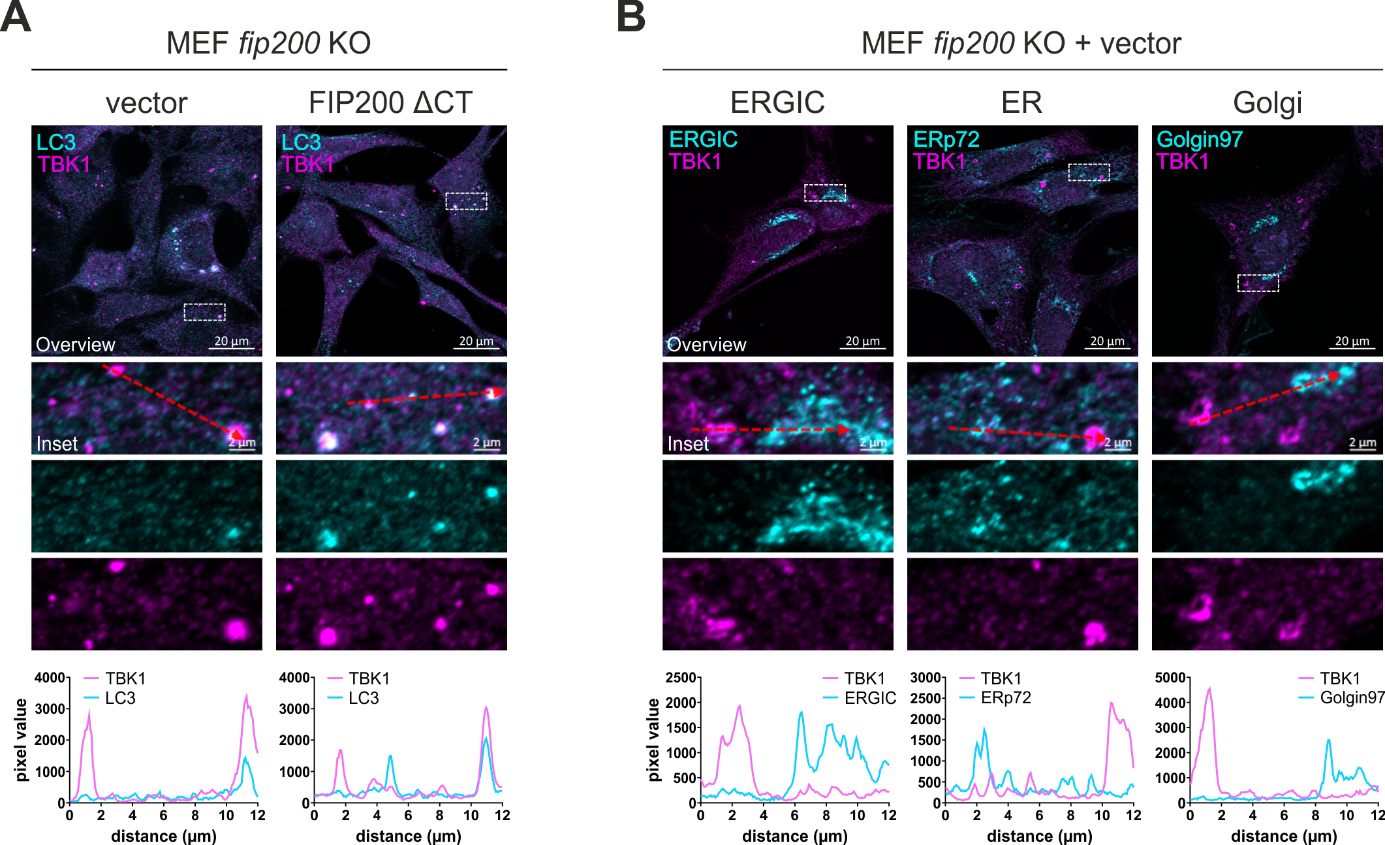
**

**Supplementary Figure S2: TBK1 aggregates partially colocalize with LC3 but do not localize to the Golgi, ER or ERGIC.** (**A**) *fip200* KO MEFs transfected with empty vector or cDNA encoding FIP200 ΔCT were fixed in 100% MeOH and immunostained for TBK1 and LC3. (**B**) Empty vector-transfected *fip200* KO MEFs were fixed in 100% MeOH and immunostained for TBK1 in combination with ERGIC, ERp72, or Golgin97. The bar graphs show the pixel intensities of the areas indicated by the respective dashed red arrows shown in the insets.

**
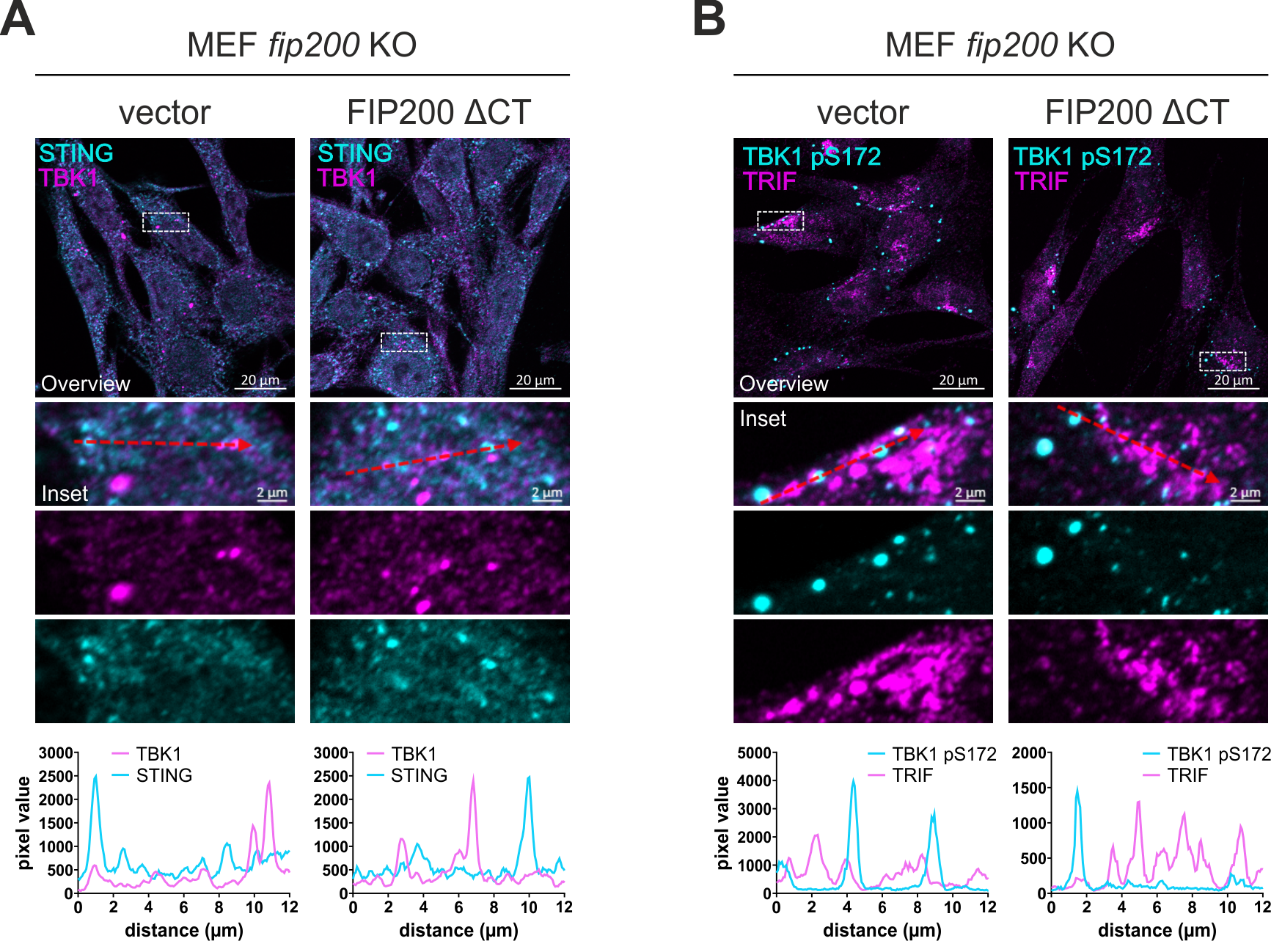
**

**Supplementary Figure S3: TBK1 aggregates do not colocalize with STING or TRIF.** (**A**) *fip200* KO MEFs transfected with empty vector or cDNA encoding FIP200 ΔCT were fixed in 100% MeOH and immunostained for TBK1 and STING. (**B**) The MEFs described in (A) were fixed in 4% PFA and immunostained for TBK1 pS172 and TRIF. The bar graphs show the pixel intensities of the areas indicated by the respective dashed red arrows shown in the insets.

**
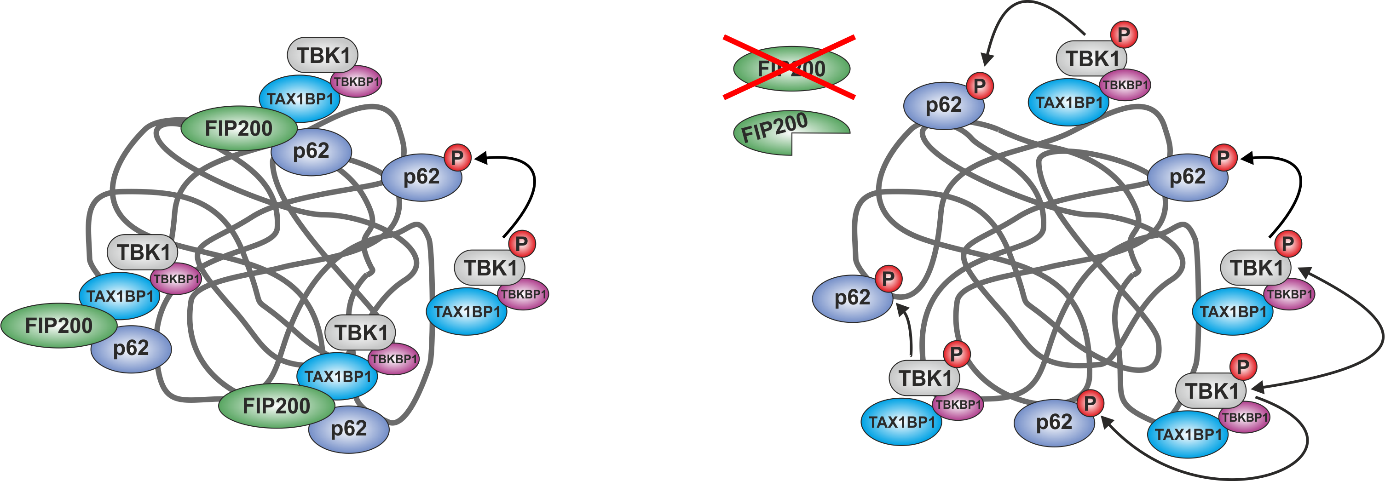
**

**Supplementary Figure S4: FIP200 controls the TBK1 activation threshold at SQSTM1/p62-positive condensates.** In FIP200-expressing cells (left), TBK1 activation at SQSTM1/p62-positive aggregates is maintained at basal levels. TBK1-dependent phosphorylation of SQSTM1/p62 at Ser403 presumably contributes to the efficient engulfment of protein aggregates. In cells deficient in FIP200 or expressing a C-terminally truncated variant of FIP200 (right), TBK1 auto-transphosphorylation and TBK1-dependent phosphorylation of SQSTM1/p62 are increased, likely contributing to the increased formation of insoluble protein aggregates. FIP200, focal adhesion kinase (FAK)-interacting protein of 200 kDa; SQSTM1/p62, sequestosome 1; TAX1BP1, Tax1-binding protein 1; TBK1, TANK-binding kinase 1; TBKBP1/SINTBAD, TBK1-binding protein 1

**
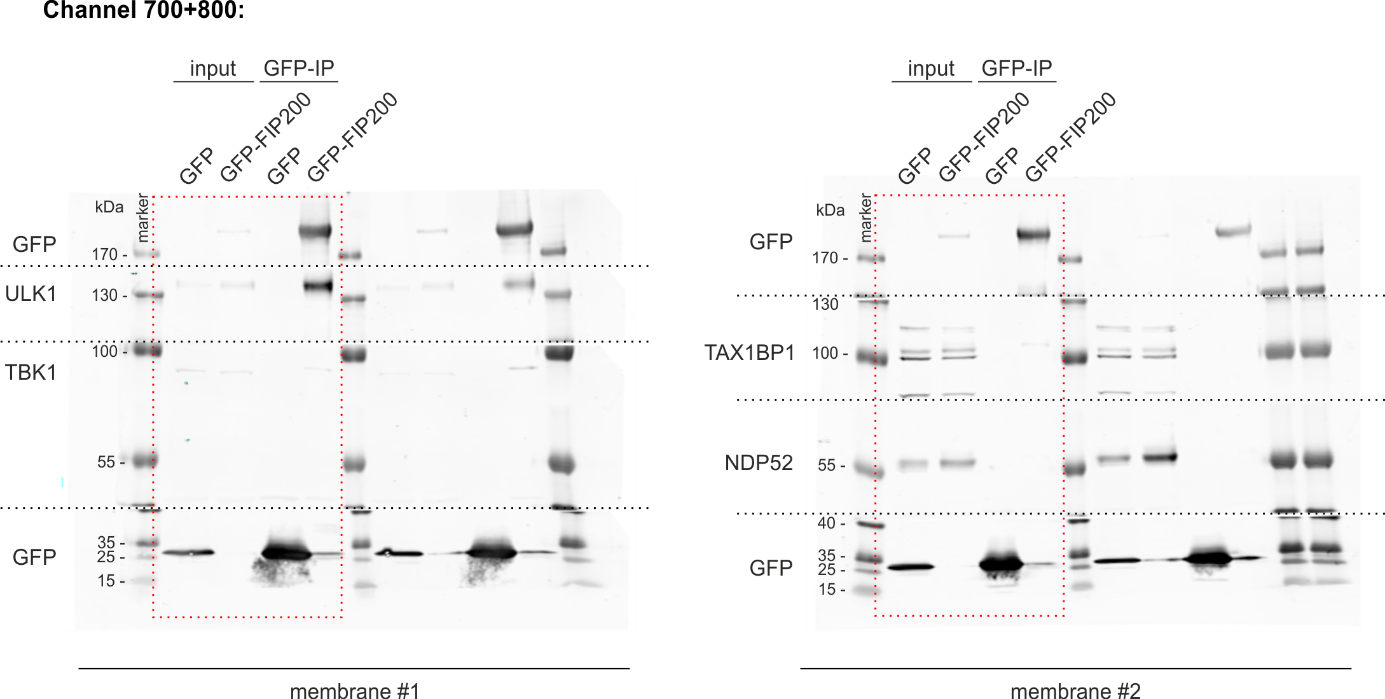
**

**Supplementary Figure S5:** **Full-length blots for the panels presented in Figure 1.** Cutting of membranes for hybridization with different antibodies is indicated by black dotted lines.

**
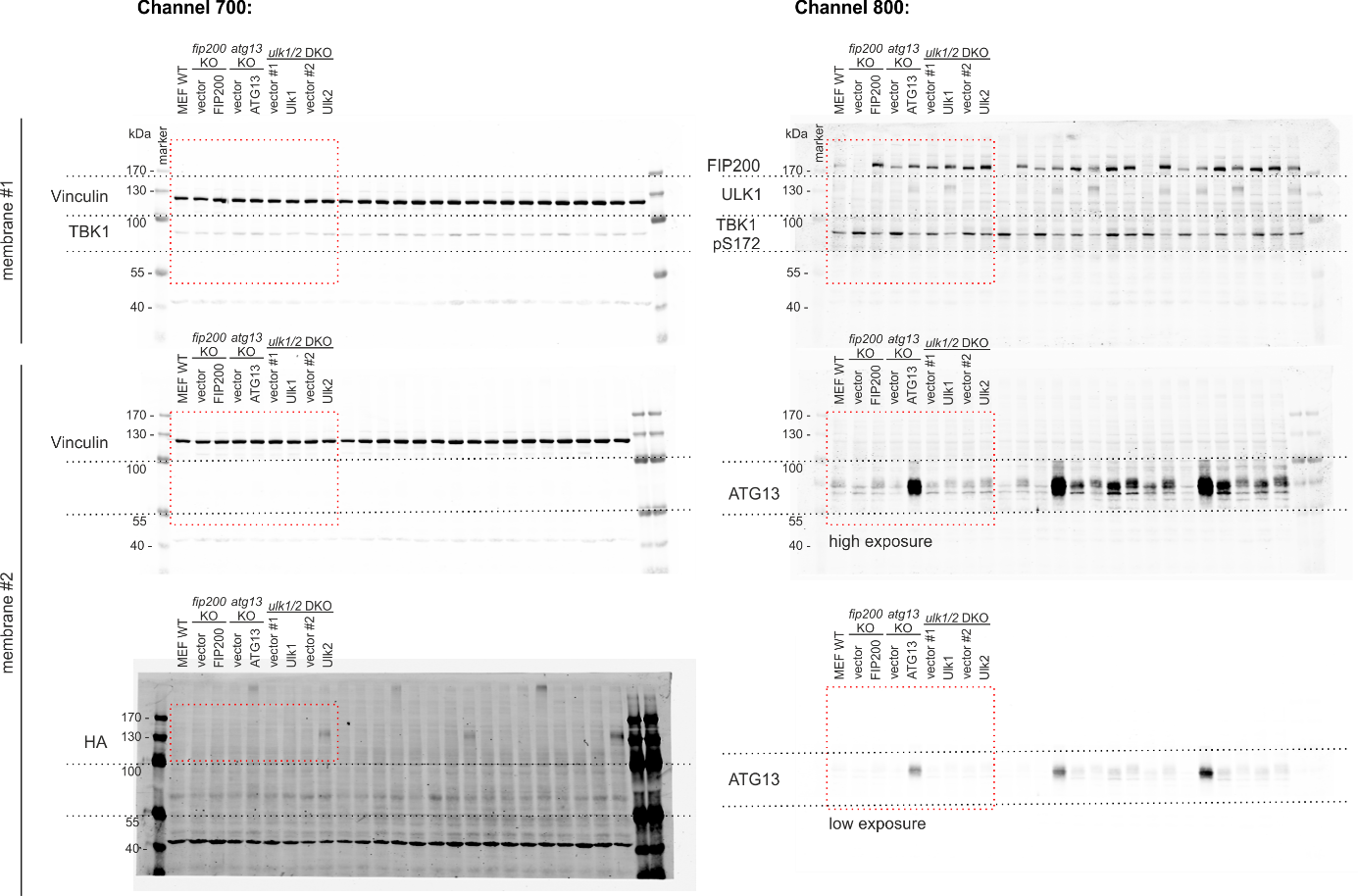
**

**Supplementary Figure S6:** **Full-length blots for the panels presented in Figure 2E.** Cutting of membranes for hybridization with different antibodies is indicated by black dotted lines.

**
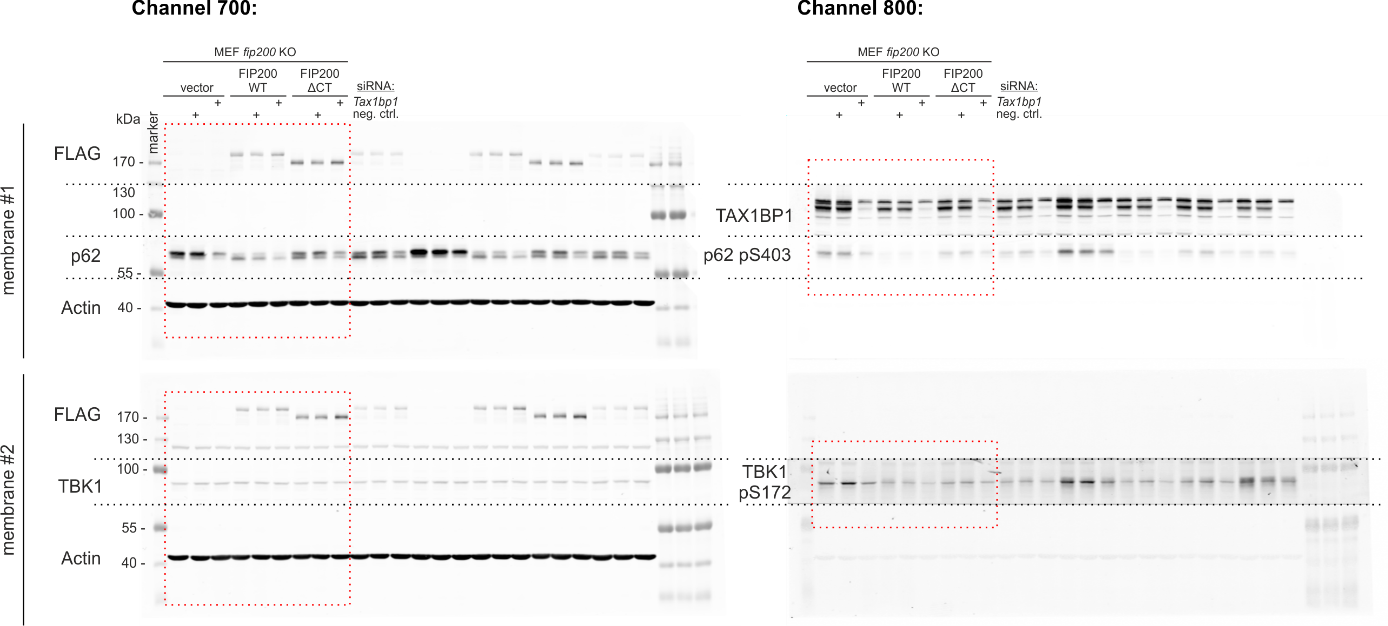
Supplementary Figure S7:** **Full-length blots for the panels presented in Figure 4A.** Cutting of membranes for hybridization with different antibodies is indicated by black dotted lines.

**
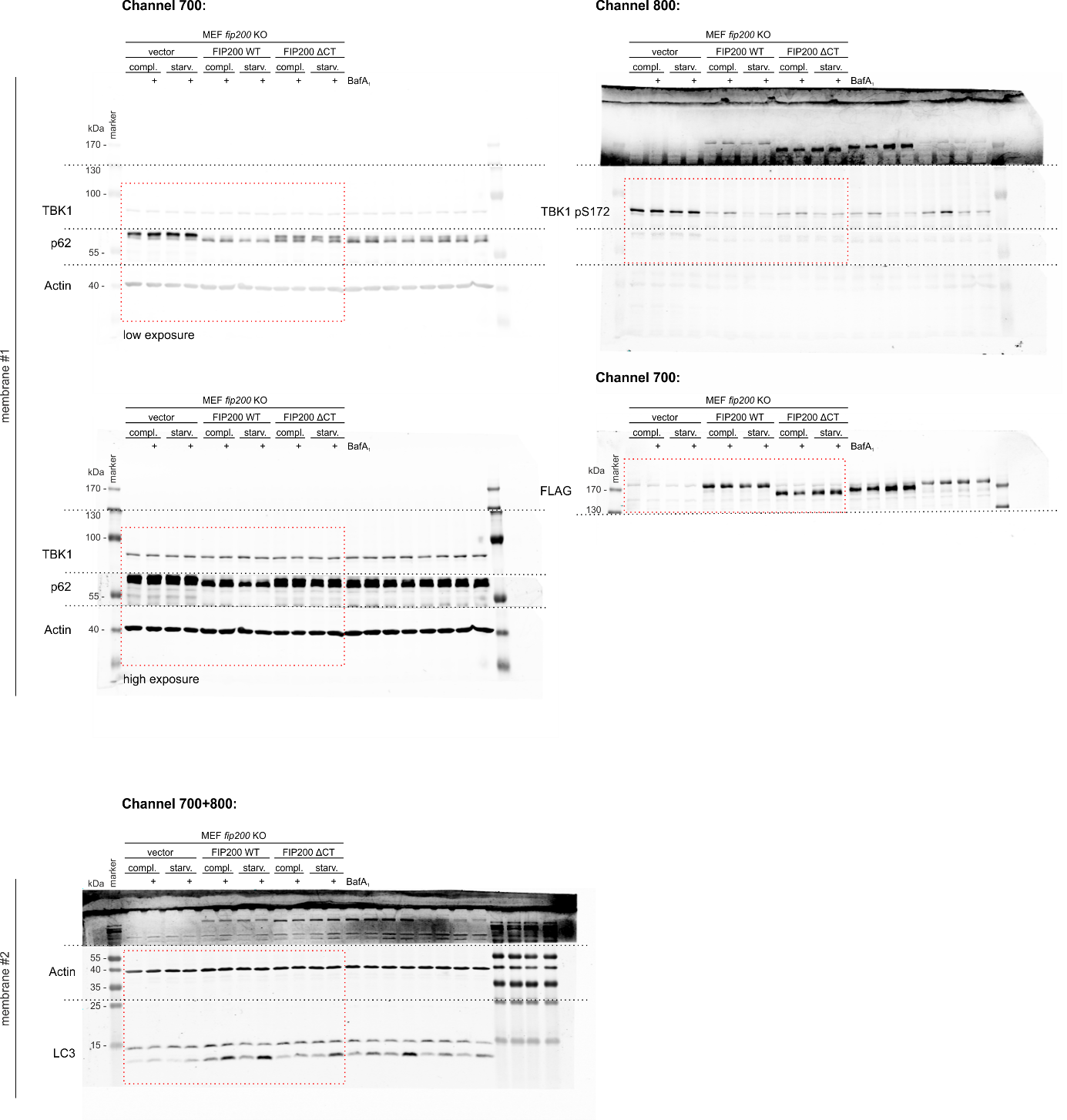
Supplementary Figure S8:** **Full-length blots for the panels presented in Figure 5A.** Cutting of membranes for hybridization with different antibodies is indicated by black dotted lines.

**
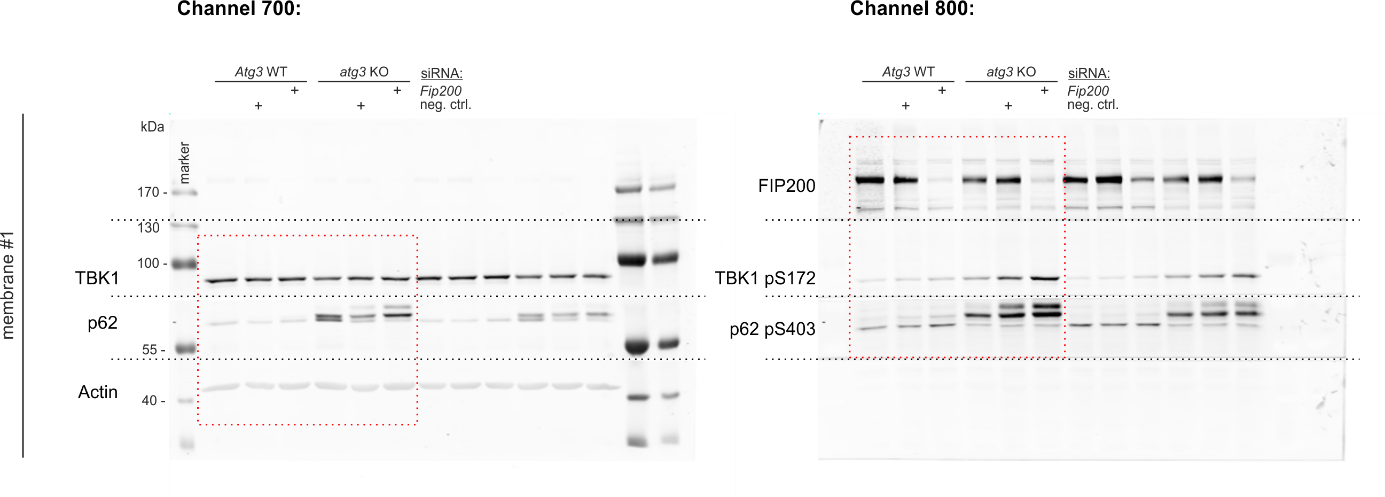
Supplementary Figure S9:** **Full-length blots for the panels presented in Figure 6A.** Cutting of membranes for hybridization with different antibodies is indicated by black dotted lines.

**
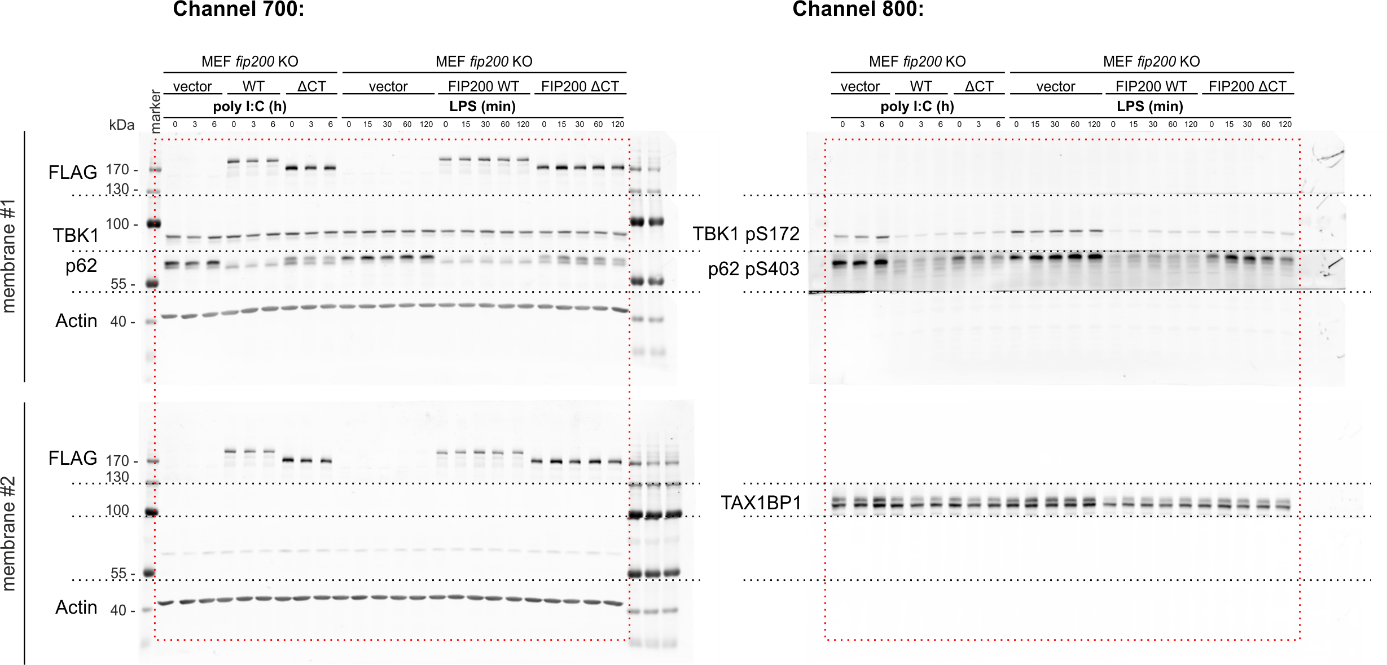
Supplementary Figure S10:** **Full-length blots for the panels presented in Figure 7B.** Cutting of membranes for hybridization with different antibodies is indicated by black dotted lines.

**
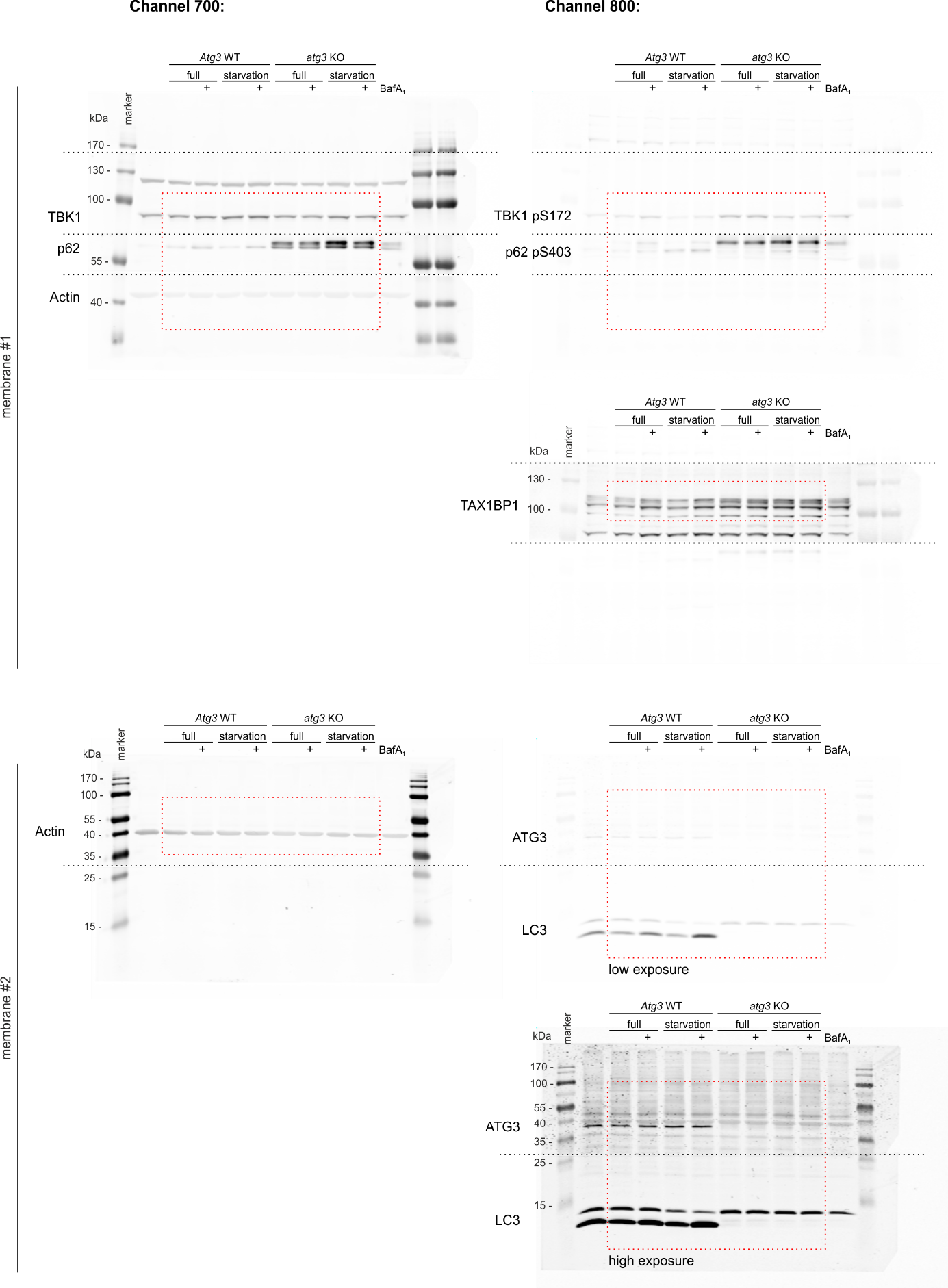
**

**Supplementary Figure S11:** **Full-length blots for the panels presented in Figure S1A.** Cutting of membranes for hybridization with different antibodies is indicated by black dotted lines.

**
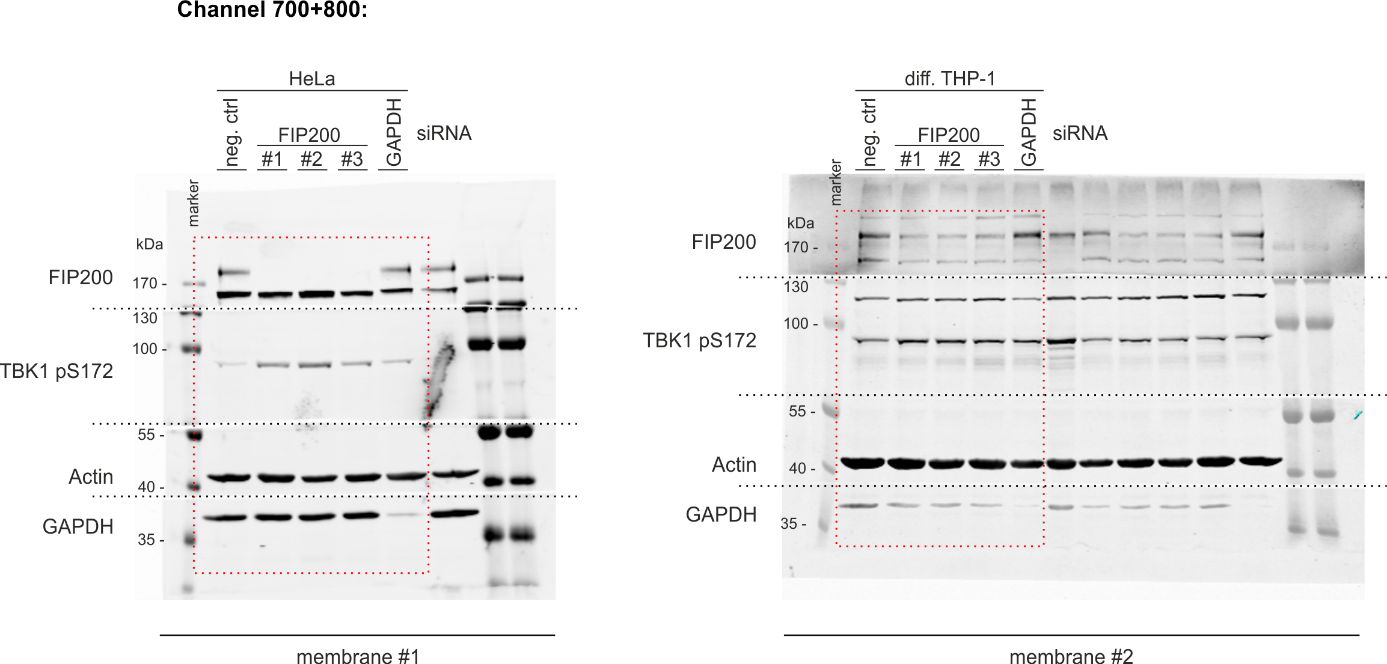
Supplementary Figure S12:** **Full-length blots for the panels presented in Figure S1B.** Cutting of membranes for hybridization with different antibodies is indicated by black dotted lines.

**Legends to Supplementary Tables S1 and S2**

**Legend to Table S1:**

**Mass spectrometric identification of GFP-FIP200-interacting proteins.** GFP or GFP-FIP200 protein was purified from the corresponding Flp-In T REx 293 cells using GFP-Trap beads. The purified proteins were analyzed by MS.

**Legend to Table S2:**

**Macro for analysis of TBK1 pS172 structures.** A macro was used to automatically analyze TBK1 pS172-positive structures using ImageJ 1.53c. The two channels, DAPI and TBK1 pS172, were first split and were then processed differently. To count the nuclei within one image, the DAPI signal was blurred, the background was subtracted, and the contrast was enhanced. An automatic threshold was then set based on the Li-White method, holes were filled, and an adjustable watershed was performed. All particles with a size of at least 30 were counted, representing the number of nuclei. To count the TBK1 pS172 structures, convoluted background subtraction was performed, and then an automatic threshold was set based on the MaxEntropy-White method. All particles with a size of at least 0.1 were counted, representing the number of TBK1 pS172 structures. To determine the area and intensity of each TBK1 pS172 structure, all counted structures were set as regions of interest (ROIs), and these ROIs were transferred to unprocessed images. Then, the area and intensity of each TBK1 pS172 structure were measured and transferred to Excel (Microsoft Corporation).
